# Invariant Measures for the Stochastic One-Dimensional Compressible Navier–Stokes Equations

**DOI:** 10.1007/s00245-019-09594-x

**Published:** 2019-07-16

**Authors:** Michele Coti Zelati, Nathan Glatt-Holtz, Konstantina Trivisa

**Affiliations:** 1 Department of Mathematics, Imperial College London, London, SW7 2AZ UK; 2 Department of Mathematics, Tulane University, New Orleans, LA 70118 USA; 3 Department of Mathematics, University of Maryland, College Park, MD 20742 USA

**Keywords:** Navier–Stokes system, Compressible fluid, Stochastic perturbation, Invariant measure, 35R60, 37L40, 35Q35, 60H30

## Abstract

We investigate the long-time behavior of solutions to a stochastically forced one-dimensional Navier–Stokes system, describing the motion of a compressible viscous fluid, in the case of linear pressure law. We prove existence of an invariant measure for the Markov process generated by strong solutions. We overcome the difficulties of working with non-Feller Markov semigroups on non-complete metric spaces by generalizing the classical Krylov–Bogoliubov method, and by providing suitable polynomial and exponential moment bounds on the solution, together with pathwise estimates.

## Introduction

We consider the question of existence of an invariant measure for the stochastically forced Navier–Stokes equations of compressible fluid flows in one space dimension. Formulated on the unit interval (0, 1), the equations read

1.1 $$\begin{aligned}&\rho _t + (\rho u)_x = 0 \end{aligned}$$

1.2 $$\begin{aligned}&\mathrm{d}(\rho u) + \left( \rho u^2 + A^2\rho \right) _x \mathrm{d}t = u_{xx} \mathrm{d}t + \rho \sigma \mathrm{d}W, \end{aligned}$$

where $\rho =\rho (t,x)$ is the fluid density and $u=u(t,x)$ is the fluid velocity. System (1.1)–(1.2) is appropriately written in dimensionless form, and is supplemented by the initial conditions

1.3 $$\begin{aligned} \rho (0,x)=\rho _0(x), \qquad u(0,x)=u_0(x), \end{aligned}$$

where $\rho _0,u_0$ are assigned functions defined on the interval [0, 1], with the (strictly positive) initial density having normalized mass

$$\begin{aligned} \int _0^1 \rho _0(x) \mathrm{d}x = 1. \end{aligned}$$

In view of mass conservation, we obviously have

1.4 $$\begin{aligned} \int _0^1 \rho (t,x) \mathrm{d}x = 1, \qquad \forall t\ge 0. \end{aligned}$$

Moreover, we assume homogeneous Dirichlet boundary conditions for the velocity *u*, namely

1.5 $$\begin{aligned} u(t,0)=u(t,1)=0, \qquad \forall t\ge 0. \end{aligned}$$

Above, the driving noise is given by a collection of independent white noise processes, suitably colored in space (see (2.1) below), while $A>0$ is a dimensionless parameter, inversely proportional to the Mach number. When considering strong (in the deterministic sense) solutions to (1.1)–(1.5), the natural phase space for our system is

1.6 $$\begin{aligned} \mathcal {X}=\left\{ (\rho ,u)\in H^1(0,1)\times H^1_0(0,1): \int _0^1 \rho (x) \mathrm{d}x = 1, \ \rho >0\right\} . \end{aligned}$$

The main result of this article is the following.

### Theorem 1.1

For every fixed $A>0$, the Markov semigroup $\{{\mathcal {P}}_t\}_{t\ge 0}$ associated to (1.1)–(1.5) possesses an invariant probability measure $\mu _A\in {\mathfrak {P}}(\mathcal {X}_{L^2})$. Furthermore,

1.7 $$\begin{aligned} \int _{\mathcal {X}} \left[ A^2\Vert (\log \rho )_x\Vert _{L^2}^2+\Vert u_x\Vert _{L^2}^2\right] \mathrm{d}\mu _A(\rho ,u) \le \Vert \sigma \Vert ^2_{L^\infty }. \end{aligned}$$

Here, $\mathcal {X}_{L^2}$ denotes the set $\mathcal {X}$ in (1.6), endowed with the $L^2\times L^2$ metric, and $\Vert \sigma \Vert _{L^\infty }$ is given by (2.2) below.

The existence of statistically stationary states to randomly driven systems in fluid dynamics is of basic importance from both mathematical and experimental viewpoints. On the one hand, the existence of an invariant measure provides information on the generic long-time behavior of the system. On the other hand, under ergodicity assumptions, it provides a link between experimental observations (for example, in turbulence theory) and theoretical predictions.

In the context of fluid dynamics, a satisfactory theory of invariant measures has been developed for two-dimensional, incompressible flows [6, 7, 14, 22, 26, 27, 36, 37, 41, 42, 52]. In three dimensions, we mention the works [11, 12, 23], and especially [21], in which solutions which are strictly stationary stochastic processes are constructed, but the concept of invariant measure as a steady state is not well defined due to the absence of the Markov property. In this sense, the situation for multi-dimensional compressible flows is similar. To the best of our knowledge, the only relevant articles are [24], in which an invariant measure is constructed for an approximation of the Lagrangian version of (1.1)–(1.2) and for a different pressure law, and the more recent [4], in which statistically stationary solutions (but no invariant measure) are constructed. There are, however, results for both the dissipative and non-dissipative one-dimensional Burgers equation [10, 13, 25, 44, 51].

In this article, we prove existence of an invariant measure for the compressible Navier–Stokes system. The main advantage in working in one space dimension is that the equations are globally well-posed and can be solved pathwise, leaning on various works in the deterministic setting [28, 29, 31–34, 43, 46]. For the global solvability in the stochastic case, we mention [48, 49], valid in one space dimension, and [2, 5, 45, 47, 50] for the multi-dimensional case. For the deterministic set-up, we refer to the classical references [16, 38], and to [18, 19] for long-time behavior results.

In turn, (1.1)–(1.5) generates a proper Markov semigroup, and the concept of an invariant measure can be defined in a standard manner. However, there are several obstructions on the path towards the proof of Theorem 1.1. The main ingredients of our approach and contributions to the existing theory on invariant measures can be summarized as follows:

- *An *$L^2$*-based continuity result via the derivation of polynomial and exponential moment bounds* As it is is clear from its statement, the topology on $\mathcal {X}$ plays an essential role. The metric space $\mathcal {X}_{L^2}$ is not complete (hence not Polish), due to both the open condition $\rho >0$ and the fact that the Sobolev space $H^1$ is not a closed subspace of $L^2$. This choice is dictated by the available continuous dependence estimates (cf. Theorem 2.7), which make use of the so-called relative entropy functional and essentially provide an $L^2$-based continuity result. Such a result crucially depends on polynomial and exponential moment bounds of the solution to (1.1)–(1.5), which are carried out in detail in Sect. 2.1, and on careful pathwise estimates (see Sect. 2.2).
- *Introduction of a (larger) class of functions on *$\mathcal {X}_{L^2}$ *which is invariant under the Markov semigroup *${\mathcal {P}}_t$ Closely related to the issue mentioned above, a second difficulty lies in the fact that the Markov semigroup associated to (1.1)–(1.5) is not known to be Feller (cf. Sect. 3.1), namely ${\mathcal {P}}_t$ may not map $C_b(\mathcal {X}_{L^2})$ (the real-valued, continuous bounded functions on $\mathcal {X}_{L^2}$) into itself. This is again attributable to the mismatch between the set $\mathcal {X}$ and the $L^2$ topology, and causes problems when trying to apply the classical Krylov–Bogoliubov procedure to prove existence and, in particular, invariance of a probability measure constructed as a subsequential limit of time-averaged measures. We circumvent this issue by defining a class of functions on $\mathcal {X}_{L^2}$ that is slightly larger that $C_b(\mathcal {X}_{L^2})$, which is invariant under the Markov semigroup ${\mathcal {P}}_t$, and still well-behaved in the above limiting procedure.
- *Derivation of lower and upper bounds of the density with the correct time-averaged growth* Lastly, the lack of instantaneous smoothing in the density equation (1.1) constitutes an obstacle towards compactness estimates for time-averaged measures. However, an energy structure already exploited in [32, 43] allows to obtain a dissipation term involving $\Vert (\log \rho )_x\Vert _{L^2}$, thanks to the linear pressure law adopted here. Consequently, upper and lower bounds on the density (which, in 1D, can be proven with more general pressure laws) have the correct time-averaged growth and provide suitable tightness estimates for time-averaged measures (cf. Sect. 3.2).
- The present article is, according to our knowledge, the first that presents rigorous results on the existence of invariant measures for compressible flows.

### Remark 1.2

(The low Mach number limit) One may be tempted to study the behavior of the measures $\mu _A$ as $A\rightarrow \infty$, in the spirit of compressible-incompressible limits [3, 15, 17, 39, 40]. From (1.7), it is clear that, as $A\rightarrow \infty$, the density component of the measures concentrates on sets such that $\log \rho =0$, namely, $\rho =1$. However, (1.1) implies that $u_x=0$, and due to the homogeneous boundary conditions (1.5), we deduce that also $u=0$. This is inconsistent with the second equation, as long as $\sigma \ne 0$. By replacing $\sigma$ with $A^{-\eta }\sigma$, for any $\eta >0$, it is easily seen from (1.7) that $\mu _A\rightarrow \delta _{(\rho =1,u=0)}$ as $A\rightarrow \infty$, essentially describing rigid body motion.

### Extensions and Further Developments

Theorem 1.1 is in fact true for pressure laws of the type

$$\begin{aligned} p(\rho )\sim \rho , \quad \text {as } \rho \rightarrow 0, \end{aligned}$$

and any polynomial growth as $\rho \rightarrow \infty$. The case of pure power law $p(\rho )=\rho ^\gamma$, for $\gamma >1$, remains open. Another important question is the uniqueness (and hence ergodicity) and the attracting properties of the measure $\mu _A$. It is worth to point out that the problem is highly degenerate, since the noise only acts on one component of the phase space. We conjecture that uniqueness holds, since the unforced system evolves (at a very slow rate) towards the unique steady state $(\rho ,u)=(1,0)$.

### Outline of the Article

Section 2 is dedicated to the properties of solutions to the compressible Navier–Stokes equations. Energy estimates and moment bounds are proven in Sect. 2.1, pathwise estimates in Sect. 2.2, while uniqueness and continuous dependence on data are discussed in Sects. 2.3 and 2.4 , respectively. In Sect. 3 we prove Theorem 1.1, setting up the Markovian framework and discussing existence of invariant measure for non-Feller Markov processes on non-complete metric spaces.

### Notation and Conventions

Throughout the paper, *c* will denote a *generic* positive constant *independent* of *A*, whose value may change even in the same line of a certain equation. In the same way, $C_1, C_2, \ldots$ denote specific large deterministic constants. We will denote by $\mathcal {X}_{L^2}$ the set $\mathcal {X}$ endowed with $L^2 \times L^2$ metric. Notice that $\mathcal {X}_{L^2}$ is clearly a metric space. When we want to indicate the set $\mathcal {X}$ endowed with the $H^1\times H^1$ metric, we write $\mathcal {X}_{H^1}$. By $\mathcal {B}(\mathcal {X}_{L^2})$, we denote the family of Borel subsets of $\mathcal {X}_{L^2}$. With the symbol $M_b(\mathcal {X}_{L^2})$ (resp. $C_b(\mathcal {X}_{L^2})$) we refer to the set of all real valued bounded measurable (resp. bounded continuous) functions on $\mathcal {X}_{L^2}$. Finally, ${\mathfrak {P}}(\mathcal {X}_{L^2})$ is the set of all probability measures on $\mathcal {X}_{L^2}$.

## Existence and Uniqueness of Solutions

In view of the particular form of the stochastic forcing in (1.2), the proof of existence and uniqueness of pathwise solutions borrows many ideas from the deterministic case. Fix a stochastic basis

$$\begin{aligned} \mathcal {S}=(\Omega , \mathcal {F}, {\mathbb {P}}, \{\mathcal {F}_t\}_{t\ge 0}, W). \end{aligned}$$

We write the noise term in (1.2) as

2.1 $$\begin{aligned} \sigma \mathrm{d}W= \sum _{k=1}^\infty \sigma _k(x)\mathrm{d}W^k(t). \end{aligned}$$

The sequence $W(t) =\{W^k(t)\}_{k\in {\mathbb {N}}}$ consists of independent copies of the standard one-dimensional Wiener process (Brownian motion). As such, for each *k*, $\mathrm{d}W^k(t)$ is formally a white noise which, in particular, is stationary in time. Throughout the article, we will assume

$$\begin{aligned} \Vert \sigma _{xx}\Vert _{L^2}^2 :=\int _0^1 \sum _{\ell =1}^\infty |(\sigma _\ell )_{xx}(x)|^2\mathrm{d}x<\infty , \qquad \sigma _\ell \in H^2\cap H^1_0. \end{aligned}$$

In particular, this implies

2.2 $$\begin{aligned} \Vert \sigma \Vert _{L^\infty }^2 :=\sup _{x\in (0,1)} \sum _{\ell =1}^\infty |\sigma _\ell (x)|^2<\infty . \end{aligned}$$

By considering the change of variable ${{\tilde{u}}}=u-\sigma W$, a pathwise approach for the existence of solutions (even in the multidimensional case) has been carried out in [20] (see also [47–49] and the more recent [5] for local existence of strong solutions). In the deterministic setting and in one space dimension, the local existence of strong solutions has been proved in [46], while their global existence can be found in [43], in the case of pressure law of the type $\rho ^\gamma$, for $\gamma >1$. That this result can be extended to our case is a consequence of the estimates provided in the work [32] on (deterministic) weak solutions and their regularization properties. We summarize these observations in the theorem below.

### Theorem 2.1

Fix a stochastic basis $\mathcal {S}=(\Omega , \mathcal {F}, {\mathbb {P}}, \{\mathcal {F}_t\}_{t\ge 0}, W)$. For any $(\rho _0,u_0)\in \mathcal {X}$, there exists a unique $(\rho (t;\rho _0), u(t;u_0))$ satisfying (1.1)–(1.5), in the time integrated sense and with the regularity

$$\begin{aligned} \rho \in L_{loc}^\infty (0,\infty ;H^1), \qquad u\in L_{loc}^\infty (0,\infty ;H_0^1)\cap L_{loc}^2(0,\infty ;H^2), \end{aligned}$$

almost surely. Moreover, the map $t\mapsto (\rho (t;\rho _0), u(t;u_0))$ is weakly continuous, and for every $t\ge 0$,

$$\begin{aligned} \rho (t)\in L^\infty \qquad \text {and}\qquad \frac{1}{\rho (t)}\in L^\infty , \end{aligned}$$

almost surely.

The bounds in the Theorem 2.1 (see also Lemma 2.2) for the density show that neither vacuum states nor concentration states can occur, no matter how large the initial datum is. This is one of several important differences between the Navier–Stokes equations and the inviscid Euler equations, for which vacuum states may in fact occur for large initial data and for certain equations of state (cf. [8, 9]). It is also relevant in this regard that solutions of the Navier–Stokes equations show certain instabilities when vacuum states are allowed (cf. Hoff and Serre [30]).

As far as the regularity of solutions is concerned, we need quantitative estimates and moment bounds that are new to the best of our knowledge. These estimates are contained in the the Propositions 2.3 and 2.4 below, and, in particular, agree with the regularity expressed in the theorem above.

### Energy Estimates

For a pair $(\rho ,u)\in \mathcal {X}$, we define the entropy function in the classical way as

$$\begin{aligned} \mathcal {H}(\rho ,u) = \int _0^1 \left( \frac{1}{2} \rho u^2 + A^2\rho \log \rho \right) \mathrm{d}x. \end{aligned}$$

In the one-dimensional setting, it turns out the strong solutions are also well-behaved with respect to the modified energy functional

$$\begin{aligned} \mathcal {E}(\rho , u) = \mathcal {H}(\rho , u) + \frac{1}{2} \int _0^1 \left( \frac{\rho _x u}{\rho } + \frac{1}{2} \frac{\rho _x^2}{\rho ^3} \right) \mathrm{d}x. \end{aligned}$$

Several quantities are controlled by $\mathcal {E}$. In particular, uniform estimates on $\mathcal {E}$ entail lower bounds on $\rho$.

#### Lemma 2.2

Assume that $(\rho ,u)\in \mathcal {X}$ are such that $\mathcal {E}(\rho ,u)<\infty$. Then

2.3 $$\begin{aligned} \mathrm{e}^{-\left[ 8\mathcal {E}(\rho ,u) \right] ^{1/2}} \le \rho (x) \le \mathrm{e}^{\left[ 8\mathcal {E}(\rho ,u) \right] ^{1/2}}, \qquad \forall x\in [0,1], \end{aligned}$$

and

2.4 $$\begin{aligned} \Vert \rho _x\Vert _{L^2}^2 \le 8\mathcal {E}(\rho ,u)\mathrm{e}^{3\left[ 8\mathcal {E}(\rho ,u) \right] ^{1/2}} . \end{aligned}$$

Moreover,

2.5 $$\begin{aligned} \Vert u\Vert ^2_{L^2} \le 2\mathcal {H}(\rho ,u ) \mathrm{e}^{\left[ 8\mathcal {E}(\rho ,u) \right] ^{1/2}}. \end{aligned}$$

#### Proof

As a preliminary observation we note that (pointwise)

2.6 $$\begin{aligned} \frac{\rho _x u}{\rho }\le \rho u^2+\frac{1}{4} \frac{\rho _x^2}{\rho ^3}. \end{aligned}$$

Moreover, since $\rho \in H^1$ and

2.7 $$\begin{aligned} \int _0^1\rho \, \mathrm{d}x=1, \end{aligned}$$

we have

$$\begin{aligned} \int _0^1\rho \log \rho \mathrm{d}x = \int _0^1\left[ \rho \log \rho -\rho +1\right] \mathrm{d}x\ge 0. \end{aligned}$$

Therefore

2.8 $$\begin{aligned} \mathcal {E}(\rho , u)&= \int _0^1 \left( \frac{1}{2} \rho u^2 +A^2\rho \log \rho +\frac{1}{2} \frac{\rho _x u}{\rho } + \frac{1}{4} \frac{\rho _x^2}{\rho ^3} \right) \mathrm{d}x\ge \frac{1}{8}\int _0^1 \frac{\rho _x^2}{\rho ^3} \mathrm{d}x. \end{aligned}$$

By continuity of $\rho$ and (2.7), we can choose $x_0\in (0,1)$ such that $\rho (x_0)=1$ and write

$$\begin{aligned} |\log \rho (x)|=\left| \int _{x_0}^x (\log \rho )_y\mathrm{d}y\right| \le \int _0^1 \frac{|\rho _y|}{\rho }\mathrm{d}y. \end{aligned}$$

Hence,

$$\begin{aligned} |\log \rho (x)|\le \int _0^1 \frac{|\rho _x|\rho ^{1/2}}{\rho ^{3/2}}\mathrm{d}x\le \left[ \int _0^1 \frac{\rho _x^2}{\rho ^3} \mathrm{d}x\right] ^{1/2}. \end{aligned}$$

Consequently,

$$\begin{aligned} \Vert \log \rho \Vert _{L^\infty } \le \left[ \int _0^1 \frac{\rho _x^2}{\rho ^3} \mathrm{d}x\right] ^{1/2}. \end{aligned}$$

From (2.8), the first bound (2.3) follows immediately. Regarding (2.4), we combine the upper bound in (2.3) with (2.8) to deduce that

$$\begin{aligned} \int _0^1 \rho _x^2\mathrm{d}x\le \Vert \rho \Vert _{L^\infty }^3\int _0^1 \frac{\rho _x^2}{\rho ^3}\mathrm{d}x\le 8\mathcal {E}(\rho ,u)\mathrm{e}^{3\left[ 8\mathcal {E}(\rho ,u) \right] ^{1/2}} \end{aligned}$$

Finally, (2.5) follows from the lower bound in (2.3). $\square$

It is fairly clear from the above estimates that if $(\rho ,u)\in \mathcal {X}$, then $\mathcal {E}(\rho ,u)<\infty$. Viceversa, if $(\rho ,u)$ are smooth functions such that $\mathcal {E}(\rho ,u)<\infty$, then $(\rho ,u)\in \mathcal {X}$. The above lemma provides a quantification of this dichotomy.

It is crucial for us to prove various estimates on strong solutions to the compressible Navier–Stokes equations, keeping particular attention to the dependence on the parameter $A>0$. We collect these estimates in the next two propositions, whose proofs are carried out in the subsequent sections.

#### Proposition 2.3

Consider a solution $(\rho ,u)$ to (1.1)–(1.5) with initial data $(\rho _0,u_0)\in \mathcal {X}$ in (1.3) such that

$$\begin{aligned} \mathcal {E}(\rho _0,u_0)<\infty . \end{aligned}$$

Then there hold the entropy inequality

2.9 $$\begin{aligned} {\mathbb {E}}\mathcal {H}(\rho ,u)(t) + {\mathbb {E}}\int _0^t \Vert u_x \Vert _{L^2}^2 \mathrm{d}s \le \mathcal {H}(\rho _0,u_0) + \frac{1}{2}\Vert \sigma \Vert ^2_{L^\infty }t, \end{aligned}$$

and the energy inequality

2.10 $$\begin{aligned}&{\mathbb {E}}\mathcal {E}(\rho ,u)(t) +\frac{1}{2}{\mathbb {E}}\int _0^t\Vert u_x\Vert ^2_{L^2}\mathrm{d}s + \frac{A^2}{2}{\mathbb {E}}\int _0^t\Vert (\log \rho )_x\Vert ^2_{L^2} \mathrm{d}s \nonumber \\&\quad \le \mathcal {E}(\rho _0,u_0) + \frac{1}{2} \Vert \sigma \Vert ^2_{L^\infty }t, \end{aligned}$$

for all $t\ge 0$. Moreover, the exponential martingale estimate

2.11 $$\begin{aligned}&{\mathbb {P}}\left[ \sup _{t\ge 0}\left( \mathcal {E}(\rho ,u)(t) + \frac{1}{4}\int _0^t\left[ \Vert u_x\Vert ^2_{L^2} +A^2\Vert (\log \rho )_x\Vert ^2_{L^2} \right] \mathrm{d}s\right. \right. \nonumber \\&\quad \left. \left. -\frac{1}{2}\Vert \sigma \Vert ^2_{L^\infty }t\right) -\mathcal {E}(\rho _0,u_0)\ge R\right] \le \mathrm{e}^{-\gamma _0 R}, \end{aligned}$$

holds for every $R\ge 0$ and for

$$\begin{aligned} \gamma _0:= \frac{\min \left\{ 1,4A^2\right\} }{4\Vert \sigma \Vert ^2_{L^\infty }}. \end{aligned}$$

As a consequence, for every $m\ge 1$, there exists a constant $c_m>0$ such that

2.12 $$\begin{aligned}&{\mathbb {E}}\sup _{t\in [0,T]} \left( \mathcal {E}(\rho ,u)(t) + \frac{1}{4}\int _0^t\left[ \Vert u_x\Vert ^2_{L^2} +A^2\Vert (\log \rho )_x\Vert ^2_{L^2} \right] \mathrm{d}s \right) ^m\nonumber \\&\quad \le c_m\left( \mathcal {E}(\rho _0,u_0)^m+\Vert \sigma \Vert ^{2m}_{L^\infty } T^m+\gamma _0^{-m}\right) , \end{aligned}$$

for every $T\ge 1$, and the exponential moment bound holds

2.13 $$\begin{aligned}&{\mathbb {E}}\exp \left( \frac{\gamma _0}{2} \sup _{t\in [0,T]} \left( \mathcal {E}(\rho ,u)(t) + \frac{1}{4}\int _0^t\left[ \Vert u_x\Vert ^2_{L^2} +A^2\Vert (\log \rho )_x\Vert ^2_{L^2} \right] \mathrm{d}s \right) \right) \nonumber \\&\quad \le \exp \frac{\gamma _0}{2} \left( \mathcal {E}(\rho _0,u_0)+\frac{1}{2}\Vert \sigma \Vert ^2_{L^\infty }T\right) , \end{aligned}$$

q for all $$T\ge 1$$.

The next result is instead a collection of similar, yet pathwise, estimates.

#### Proposition 2.4

Consider a solution $(\rho ,u)$ to (1.1)–(1.5) with initial data $(\rho _0,u_0)\in \mathcal {X}$ such that $\mathcal {E}(\rho _0,u_0)<\infty$. For every $T\ge 0$ and almost surely, there holds

2.14 $$\begin{aligned}&\sup _{t\in [0,T]}\left[ \mathcal {E}(\rho (t),u(t))+\int _0^T\left[ \Vert u_x\Vert _{L^2}^2 + A^2 \Vert (\log \rho )_x\Vert _{L^2}^2\right] \mathrm{d}t\right] \nonumber \\&\quad \le C_1(\sigma W,A, \mathcal {E}(\rho _0,u_0),T). \end{aligned}$$

In particular,

2.15 $$\begin{aligned}&\sup _{t\in [0,T]}\left[ \Vert \rho (t)\Vert _{L^\infty }+\Vert \rho ^{-1}(t)\Vert _{L^\infty }\right] \nonumber \\&\quad \le C_2(\sigma W,A, \mathcal {E}(\rho _0,u_0),T). \end{aligned}$$

Moreover,

2.16 $$\begin{aligned} \sup _{t\in [0,T]}\left[ \Vert u_x(t)\Vert _{L^2}^2+\int _0^T\left[ \int _0^1\frac{|u_{xx}|^2}{\rho }\mathrm{d}x\right] \mathrm{d}t\right] \le C_3(\sigma W,A, \mathcal {E}(\rho _0,u_0),\Vert (u_0)_x\Vert _{L^2},T). \end{aligned}$$

In the above the constants $C_i$ can be explicitly computed.

The proof of (2.15) is simply a consequence of (2.3). We begin with Proposition 2.3.

#### Entropy Estimates

According to the Itō lemma

$$\begin{aligned} \mathrm{d}( \rho u^2)&= \mathrm{d}\left( \frac{1}{\rho } (\rho u) ^2\right) = - \rho _t u^2\mathrm{d}t + \frac{1}{\rho }( 2 \rho u \cdot \mathrm{d}(\rho u) + \mathrm{d}(\rho u) \mathrm{d}(\rho u) ) \\&= (\rho u)_x u^2 \mathrm{d}t + 2 u \left[ u_{xx} - \left( \rho u^2 +A^2\rho \right) _x \right] \mathrm{d}t \\&\quad + 2 \sum _{\ell =1}^\infty \rho u \sigma _\ell \mathrm{d}W^\ell + \sum _{\ell =1}^\infty \rho |\sigma _\ell |^2 \mathrm{d}t. \end{aligned}$$

Hence, integrating by parts,

$$\begin{aligned}&\mathrm{d}\frac{1}{2}\int _0^1\rho u^2 \mathrm{d}x + \Vert u_x\Vert _{L^2}^2\mathrm{d}t = - A^2\int _0^1\rho _x u\mathrm{d}x \mathrm{d}t \\&\quad + \sum _{\ell =1}^\infty \left( \int _0^1 \rho u \sigma _\ell \mathrm{d}x \right) \mathrm{d}W^\ell + \frac{1}{2}\sum _{\ell =1}^\infty \int _0^1\rho |\sigma _\ell |^2 \mathrm{d}x \mathrm{d}t. \end{aligned}$$

Moreover, by integration by parts and using only (1.1), we have

$$\begin{aligned} \frac{\mathrm{d}}{\mathrm{d}t}\int _0^1 \rho \log \rho \mathrm{d}x = -\int _0^1 (\rho u)_x \left[ \log \rho +1\right] \mathrm{d}x= \int _0^1 \rho _x u \mathrm{d}x. \end{aligned}$$

Combining the above computations we find

2.17 $$\begin{aligned} \mathrm{d}\mathcal {H}(\rho ,u)+ \Vert u_x\Vert _{L^2}^2\mathrm{d}t = \sum _{\ell =1}^\infty \left( \int _0^1 \rho u \sigma _\ell \mathrm{d}x \right) \mathrm{d}W^\ell + \frac{1}{2}\sum _{\ell =1}^\infty \int _0^1\rho |\sigma _\ell |^2 \mathrm{d}x \mathrm{d}t, \end{aligned}$$

so that, for every $t\ge 0$, we find the entropy balance

$$\begin{aligned} {\mathbb {E}}\mathcal {H}(\rho ,u)(t) + {\mathbb {E}}\int _0^t \Vert u_x \Vert _{L^2}^2 \mathrm{d}s = \mathcal {H}(\rho _0,u_0) + \frac{1}{2}{\mathbb {E}}\sum _{\ell =1}^\infty \int _0^t\int _0^1\rho |\sigma _\ell |^2 \mathrm{d}x \mathrm{d}s. \end{aligned}$$

Notice that by (1.4) and (2.2), we have

$$\begin{aligned} \sum _{\ell =1}^\infty \int _0^t\int _0^1\rho |\sigma _\ell |^2 \mathrm{d}x \mathrm{d}s\le \Vert \sigma \Vert ^2_{L^\infty }t, \end{aligned}$$

so that

$$\begin{aligned} {\mathbb {E}}\mathcal {H}(\rho ,u)(t) + {\mathbb {E}}\int _0^t \Vert u_x \Vert _{L^2}^2 \mathrm{d}s \le \mathcal {H}(\rho _0,u_0) + \frac{1}{2}\Vert \sigma \Vert ^2_{L^\infty }t. \end{aligned}$$

This is precisely (2.9).

#### Energy Estimates

Let $\ell = \rho ^{-1/2}$ so that

$$\begin{aligned}&\ell _x = - \frac{1}{2} \frac{\rho _x}{\rho ^{3/2}}, \qquad \ell _x^2 = \frac{1}{4} \frac{\rho _x^2}{\rho ^3},\\&(\ell _x)_t = -\frac{1}{2} \frac{(\rho _x)_t}{\rho ^{3/2}} + \frac{3}{4} \frac{\rho _x \rho _t}{\rho ^{5/2}} = \frac{1}{2} \frac{(\rho u)_{xx}}{\rho ^{3/2}} - \frac{3}{4} \frac{\rho _x (\rho u)_x}{\rho ^{5/2}},\\&\ell _{xx} = -\frac{1}{2} \frac{\rho _{xx}}{\rho ^{3/2}} + \frac{3}{4} \frac{\rho _x^2}{\rho ^{5/2}}. \end{aligned}$$

As such

$$\begin{aligned} (\ell _x^2)_t + (\ell _x^2 u)_x&= - \frac{\rho _x}{\rho ^{3/2}} ( (\ell _x)_t + \ell _{xx} u) + \ell _x^2 u_x\\&= -\frac{\rho _x}{\rho ^{3/2}} \left( \frac{1}{2} \frac{2 \rho _x u_x + \rho u_{xx}}{\rho ^{3/2}} - \frac{3}{4} \frac{\rho _x \rho u_x}{\rho ^{5/2}} \right) + \frac{1}{4} \frac{\rho _x^2 u_x}{\rho ^3} \\&= - \frac{1}{2} \frac{\rho _x u_{xx}}{\rho ^{2}}. \end{aligned}$$

Notice that this computation only makes use of (1.1). On the other hand

$$\begin{aligned} \mathrm{d}\left( \frac{\rho _x u}{\rho }\right)&= \rho u \left( \frac{\rho _x }{\rho ^2} \right) _t \mathrm{d}t+ \frac{\rho _x }{\rho ^2} \mathrm{d}(\rho u) \\&= \rho u \left( \frac{(\rho _x)_t }{\rho ^2} - 2 \frac{ \rho _x \rho _t}{\rho ^3} \right) \mathrm{d}t + \frac{\rho _x }{\rho ^2} \left( u_{xx} - \left( \rho u^2 + A^2\rho \right) _x \right) \mathrm{d}t \\&\quad + \sum _{\ell =1}^\infty \frac{\rho _x }{\rho } \sigma _\ell \mathrm{d}W^\ell \\&= \frac{\rho _x u_{xx} }{\rho ^2} \mathrm{d}t - \left( \frac{u(\rho u)_{xx} }{\rho } - 2 \frac{ u \rho _x (\rho u)_x }{\rho ^2} + \frac{\rho _x \left( \rho u^2 + A^2\rho \right) _x}{\rho ^2} \right) \mathrm{d}t \\&\quad + \sum _{\ell =1}^\infty \frac{\rho _x }{\rho } \sigma _\ell \mathrm{d}W^\ell \\&= \frac{\rho _x u_{xx} }{\rho ^2} \mathrm{d}t - \left( (u u_x)_x - u_x^2 + \frac{ 2 \rho _x u u_x }{\rho } - \frac{\rho _x^2 u^2}{\rho ^2} + \frac{u^2 \rho _{xx}}{\rho } + A^2\frac{ \rho _x^2}{\rho ^2}\right) \mathrm{d}t \\&\quad + \sum _{\ell =1}^\infty \frac{\rho _x }{\rho } \sigma _\ell \mathrm{d}W^\ell \\&= \frac{\rho _x u_{xx} }{\rho ^2} \mathrm{d}t - \left( (u u_x)_x - u_x^2 + \left( \frac{\rho _x u^2 }{\rho } \right) _x + A^2\frac{\rho _x^2}{\rho ^2}\right) \mathrm{d}t \\&\quad + \sum _{\ell =1}^\infty \frac{\rho _x }{\rho } \sigma _\ell \mathrm{d}W^\ell . \end{aligned}$$

Combining the above two equations and weighting appropriately we infer

$$\begin{aligned} \mathrm{d}\left( \frac{\rho _x u}{\rho } + 2 \ell _x^2 \right) + \left( \frac{\rho _x u^2 }{\rho } +u u_x+ 2 \ell _x^2 u \right) _x \mathrm{d}t + A^2\frac{\rho _x^2}{\rho ^2} \mathrm{d}t = u_x^2 \mathrm{d}t + \sum _{\ell =1}^\infty \frac{\rho _x }{\rho } \sigma _\ell \mathrm{d}W^\ell , \end{aligned}$$

and hence

2.18 $$\begin{aligned} \mathrm{d}\int _0^1\left( \frac{\rho _x u}{\rho } + \frac{1}{2} \frac{\rho _x^2}{\rho ^3} \right) \mathrm{d}x +A^2\int _0^1 \frac{\rho _x^2}{\rho ^2} \mathrm{d}x \mathrm{d}t = \Vert u_x\Vert ^2_{L^2} \mathrm{d}t+ \sum _{\ell =1}^\infty \left( \int _0^1 \frac{\rho _x }{\rho } \sigma _\ell \mathrm{d}x \right) \mathrm{d}W^\ell . \end{aligned}$$

We sum together (2.17) and half of (2.18) to finally obtain

2.19 $$\begin{aligned}&\mathrm{d}\mathcal {E}(\rho ,u) + \frac{1}{2}\Vert u_x\Vert ^2_{L^2}\mathrm{d}t + \frac{A^2}{2}\int _0^1 \frac{\rho _x^2}{\rho ^2} \mathrm{d}x \mathrm{d}t\nonumber \\&\quad = \sum _{\ell =1}^\infty \left( \int _0^1 \left( \rho u + \frac{1}{2} \frac{\rho _x }{\rho } \right) \sigma _\ell \mathrm{d}x\right) \mathrm{d}W^\ell \nonumber \\&\qquad + \frac{1}{2} \sum _{\ell =1}^\infty \int _0^1\rho |\sigma _\ell |^2 \mathrm{d}x \mathrm{d}t. \end{aligned}$$

Hence

$$\begin{aligned}&{\mathbb {E}}\mathcal {E}(\rho ,u)(t) +\frac{1}{2}{\mathbb {E}}\int _0^t\Vert u_x\Vert ^2_{L^2}\mathrm{d}s + \frac{A^2}{2}{\mathbb {E}}\int _0^t\int _0^1 \frac{\rho _x^2}{\rho ^2} \mathrm{d}x \mathrm{d}s\\&\quad = \mathcal {E}(\rho _0,u_0) + \frac{1}{2} {\mathbb {E}}\sum _{\ell =1}^\infty \int _0^t \int _0^1\rho |\sigma _\ell |^2 \mathrm{d}x \mathrm{d}s, \end{aligned}$$

so that

$$\begin{aligned} {\mathbb {E}}\mathcal {E}(\rho ,u)(t) +\frac{1}{2}{\mathbb {E}}\int _0^t\Vert u_x\Vert ^2_{L^2}\mathrm{d}s + \frac{A^2}{2}{\mathbb {E}}\int _0^t\int _0^1 \frac{\rho _x^2}{\rho ^2} \mathrm{d}x \mathrm{d}s \le \mathcal {E}(\rho _0,u_0) + \frac{1}{2} \Vert \sigma \Vert ^2_{L^\infty }t, \end{aligned}$$

and therefore (2.10) holds.

#### Exponential Estimates

It remains to prove (2.11), which follows from (2.19) and an application of the exponential martingale estimate

2.20 $$\begin{aligned} {\mathbb {P}}\left[ \sup _{t\ge 0}\left( Z(t) - \frac{\gamma }{2} \langle Z\rangle (t)\right) \ge R\right] \le \mathrm{e}^{-\gamma R}, \qquad \forall R,\gamma >0, \end{aligned}$$

valid for any continuous martingale $\{Z(t)\}_{t\ge 0}$ with quadratic variation $\langle Z\rangle (t)$. Indeed, consider the time integrated version of (2.19), namely

2.21 $$\begin{aligned}&\mathcal {E}(\rho ,u)(t) + \frac{1}{2}\int _0^t\Vert u_x\Vert ^2_{L^2}\mathrm{d}s + \frac{A^2}{2}\int _0^t\Vert (\log \rho )_x\Vert ^2_{L^2} \mathrm{d}s \nonumber \\&\quad = \mathcal {E}(\rho _0,u_0) +Z(t)+ \frac{1}{2} \sum _{\ell =1}^\infty \int _0^t\int _0^1\rho |\sigma _\ell |^2 \mathrm{d}x \mathrm{d}s. \end{aligned}$$

where *Z*(*t*) is the martingale

$$\begin{aligned} Z(t)=\sum _{\ell =1}^\infty \int _0^t\left( \int _0^1 \left( \rho u + \frac{1}{2} \frac{\rho _x }{\rho } \right) \sigma _\ell \mathrm{d}x\right) \mathrm{d}W^\ell \end{aligned}$$

with quadratic variation

$$\begin{aligned} \langle Z\rangle (t)=\sum _{\ell =1}^\infty \int _0^t \left[ \int _0^1 \left( \rho u + \frac{1}{2} \frac{\rho _x }{\rho } \right) \sigma _\ell \mathrm{d}x\right] ^2 \mathrm{d}s. \end{aligned}$$

In view of the Poincaré-like inequality [32]

2.22 $$\begin{aligned} \int _0^1 \rho u^2\mathrm{d}x \le \Vert u_x\Vert _{L^2}^2, \end{aligned}$$

the mass constraint (1.4) and (2.2), we have

$$\begin{aligned} \sum _{\ell =1}^\infty \int _0^t \left[ \int _0^1 \rho u \sigma _\ell \mathrm{d}x\right] ^2 \mathrm{d}s \le \Vert \sigma \Vert _{L^\infty }^2\int _0^t \Vert u_x\Vert _{L^2}^2\mathrm{d}s \end{aligned}$$

and

$$\begin{aligned} \frac{1}{4}\sum _{\ell =1}^\infty \int _0^t \left[ \int _0^1 \frac{\rho _x }{\rho } \sigma _\ell \mathrm{d}x\right] ^2 \mathrm{d}s\le \frac{1}{4}\Vert \sigma \Vert _{L^\infty }^2\int _0^t \Vert (\log \rho )_x\Vert _{L^2}^2\mathrm{d}s. \end{aligned}$$

As a consequence, the quadratic variation of *Z*(*t*) can be estimated as

2.23 $$\begin{aligned} \langle Z\rangle (t)\le 2 \Vert \sigma \Vert ^2_{L^\infty } \int _0^t \left[ \Vert u_x\Vert _{L^2}^2+\frac{1}{4}\Vert (\log \rho )_x\Vert _{L^2}^2 \right] \mathrm{d}s. \end{aligned}$$

Now, from (2.21) we infer that the functional

$$\begin{aligned} \Psi (t)=\mathcal {E}(\rho ,u)(t) + \frac{1}{4}\int _0^t\Vert u_x\Vert ^2_{L^2}\mathrm{d}s + \frac{A^2}{4}\int _0^t\Vert (\log \rho )_x\Vert ^2_{L^2} \mathrm{d}s \end{aligned}$$

satisfies with probability one the inequality

2.24 $$\begin{aligned} \Psi (t)-\frac{1}{2}\Vert \sigma \Vert ^2_{L^\infty }t&\le \mathcal {E}(\rho _0,u_0)+ \left[ Z(t)- \frac{\gamma _0}{2} \langle Z\rangle (t)\right] \nonumber \\&\quad + \frac{\gamma _0}{2} \langle Z\rangle (t)- \frac{1}{4}\int _0^t\Vert u_x\Vert ^2_{L^2}\mathrm{d}s - \frac{A^2}{4}\int _0^t\Vert (\log \rho )_x\Vert ^2_{L^2} \mathrm{d}s \end{aligned}$$

where we conveniently fix the constant $\gamma _0>0$ as

$$\begin{aligned} \gamma _0:= \frac{\min \left\{ 1,4A^2\right\} }{4\Vert \sigma \Vert ^2_{L^\infty } }. \end{aligned}$$

In this way, from (2.23) it follows that

$$\begin{aligned} \frac{\gamma _0}{2} \langle Z\rangle (t)- \frac{1}{4}\int _0^t\Vert u_x\Vert ^2_{L^2}\mathrm{d}s - \frac{A^2}{4}\int _0^t\Vert (\log \rho )_x\Vert ^2_{L^2} \mathrm{d}s \le 0 \end{aligned}$$

and thus (2.24) implies

2.25 $$\begin{aligned} \Psi (t)-\frac{1}{2}\Vert \sigma \Vert ^2_{L^\infty }t\le \mathcal {E}(\rho _0,u_0)+ \left[ Z(t)- \frac{\gamma _0}{2} \langle Z\rangle (t)\right] . \end{aligned}$$

In turn, from (2.20) and the above (2.25) we deduce that

2.26 $$\begin{aligned}&{\mathbb {P}}\left[ \sup _{t\ge 0}\left( \Psi (t)-\frac{1}{2}\Vert \sigma \Vert ^2_{L^\infty }t\right) -\mathcal {E}(\rho _0,u_0)\ge R\right] \nonumber \\&\quad \le {\mathbb {P}}\left[ \sup _{t\ge 0}\left( Z(t)- \frac{\gamma _0}{2} \langle Z\rangle (t)\right) \ge R\right] \le \mathrm{e}^{-\gamma _0 R}, \end{aligned}$$

for every $R\ge 0$. This is exactly (2.11).

#### Polynomial and Exponential Moments

We now focus on (2.12), which is, in fact, an easy consequence of (2.11). Indeed, (2.26) implies that

2.27 $$\begin{aligned} {\mathbb {P}}\left[ \sup _{t\in [0,T]}\Psi (t) - \mathcal {E}(\rho _0,u_0)-\frac{1}{2}\Vert \sigma \Vert ^2_{L^\infty }T\ge R \right] \le \mathrm{e}^{-\gamma _0 R}, \end{aligned}$$

where $\Psi$ is the functional defined in (2.24). Using that, for a non-negative random variable *Z*, any $m\ge 1$ and any constant $c>0$, there holds

$$\begin{aligned}&{\mathbb {E}}[Z^m]\le 2^{m-1}{\mathbb {E}}[(Z-c)^m 1 \! \! 1_{Z>c}]+ 2^{m-1}c^m \\&\quad = 2^{m-1} \int _0^\infty {\mathbb {P}}[Z-c> \lambda ^{1/m}] \mathrm{d}\lambda + 2^{m-1}c^m, \end{aligned}$$

we infer from (2.27) that for some constant $c_m>0$, independent of $\gamma _0, T$ and $\mathcal {E}(\rho _0,u_0)$ there holds

$$\begin{aligned} {\mathbb {E}}\left[ \sup _{t\in [0,T]}\Psi (t)^m\right]&\le 2^{m-1}\int _0^\infty \mathrm{e}^{-\gamma _0 \lambda ^{1/m}}\mathrm{d}\lambda + 2^{m-1}\left( \mathcal {E}(\rho _0,u_0)+\frac{1}{2}\Vert \sigma \Vert ^2_{L^\infty }T\right) ^m\\&\le c_m\left( \gamma _0^{-m}+\mathcal {E}(\rho _0,u_0)^m+\Vert \sigma \Vert ^{2m}_{L^\infty } T^m\right) , \end{aligned}$$

which is precisely (2.12). For (2.13), the idea is similar. Indeed, for a non-negative random variable *Z* and any $\delta ,c>0$, we also have that that

$$\begin{aligned} {\mathbb {E}}[\mathrm{e}^{\delta Z}]\le \mathrm{e}^{\delta c}{\mathbb {E}}\left[ \mathrm{e}^{\delta (Z-c)1 \! \! 1_{Z>c}}\right] = \mathrm{e}^{\delta c}\int _1^\infty {\mathbb {P}}\left[ Z-c> \frac{\ln \lambda }{\delta }\right] \mathrm{d}\lambda . \end{aligned}$$

Hence, for any $\delta <\gamma _0$, we deduce from (2.27) that

$$\begin{aligned} {\mathbb {E}}\left[ \exp \left( \delta \sup _{t\in [0,T]}\Psi (t)\right) \right]\le & {} \exp \left[ \delta \left( \mathcal {E}(\rho _0,u_0)+\frac{1}{2}\Vert \sigma \Vert ^2_{L^\infty }T\right) \right] \int _1^\infty \frac{1}{\lambda ^{\gamma _0/\delta }}\mathrm{d}\lambda \nonumber \\= & {} \frac{\delta }{\gamma _0-\delta }\exp \left[ \delta \left( \mathcal {E}(\rho _0,u_0)+\frac{1}{2}\Vert \sigma \Vert ^2_{L^\infty }T\right) \right] , \end{aligned}$$

and (2.13) simply follows by choosing $\delta =\gamma _0/2$. We concluded the proof of Proposition 2.3.

### Pathwise Estimates

By setting ${{\tilde{u}}}=u-w$, with $w=\sigma W$ we obtain from (1.1) to (1.2) that the pair $(\rho ,{{\tilde{u}}})$ satisfies the system

2.28 $$\begin{aligned}&\rho _t + (\rho {{\tilde{u}}})_x = f(\rho ,w) \nonumber \\&(\rho {{\tilde{u}}})_t + ( \rho {{\tilde{u}}}^2 + A^2\rho )_x = {{\tilde{u}}}_{xx}+{{\tilde{u}}}f(\rho ,w)+\rho g(\rho ,{{\tilde{u}}},w), \end{aligned}$$

where

2.29 $$\begin{aligned} f(\rho ,w)=-(\rho w)_x, \qquad g(\rho ,{{\tilde{u}}},w)=\frac{w_{xx}}{\rho } -({{\tilde{u}}}w)_x-ww_x, \end{aligned}$$

with initial datum $(\rho _0,{{\tilde{u}}}_0)=(\rho _0,u_0)$. Notice that from (2.28) we can also write the velocity equation alone as

2.30 $$\begin{aligned} {{\tilde{u}}}_t + {{\tilde{u}}}{{\tilde{u}}}_x + A^2\frac{\rho _x}{\rho } = \frac{{{\tilde{u}}}_{xx}}{\rho }+ g(\rho ,{{\tilde{u}}},w), \end{aligned}$$

which will also be useful in the sequel. Without explicit reference, we will use (2.2) multiple times to bound the various norms of *w* appearing in the estimates.

#### Pathwise Energy Estimates

We begin by proving (2.14). A lengthy calculation analogous to that in Sect. 2.1.2 shows that

$$\begin{aligned} \frac{\mathrm{d}}{\mathrm{d}t} \mathcal {E}(\rho ,{{\tilde{u}}}) + \frac{1}{2} \Vert {{\tilde{u}}}_x\Vert _{L^2}^2 + \frac{A^2}{2} \Vert (\log \rho )_x\Vert _{L^2}^2= {\mathfrak {F}} (\rho ,{{\tilde{u}}}, f,g), \end{aligned}$$

where

$$\begin{aligned} {\mathfrak {F}}(\rho ,{{\tilde{u}}}, f,g)&= \int _0^1 \left( \frac{1}{2} f {{\tilde{u}}}^2 + A^2 f (\log \rho + 1) + \frac{1}{2} \frac{\rho _x }{\rho ^2}f {{\tilde{u}}}\right. \\&\quad \left. + \frac{1}{2}\frac{f_x {{\tilde{u}}}}{\rho } - \frac{\rho _x {{\tilde{u}}}f }{\rho ^2} + \frac{1}{2}\frac{\rho _x f_x }{\rho ^3} - \frac{3}{4}\frac{(\rho _x)^2 f}{\rho ^4} \right) \mathrm{d}x \\&\quad + \int _0^1 \left( \rho {{\tilde{u}}}g + \frac{1}{2} \frac{\rho _x }{\rho } g \right) \mathrm{d}x. \end{aligned}$$

Recalling (2.29), we may rewrite the above term as

$$\begin{aligned} {\mathfrak {F}}(\rho ,{{\tilde{u}}}, f,g)&= -A^2\int _0^1(\rho w)_x \log \rho \,\mathrm{d}x -\frac{1}{2}\int _0^1 \left[ \frac{\rho _x (\rho w)_{xx} }{\rho ^3} - \frac{3}{2}\frac{(\rho _x)^2 (\rho w)_x}{\rho ^4} \right. \nonumber \\&\quad \left. - \frac{\rho _x }{\rho } \left( \frac{w_{xx}}{\rho }-ww_x\right) \right] \mathrm{d}x \\&\quad -\frac{1}{2}\int _0^1 \left[ \left( {{\tilde{u}}}^2 - \frac{\rho _x {{\tilde{u}}}}{\rho ^2}\right) (\rho w)_x +\frac{(\rho w)_{xx} {{\tilde{u}}}}{\rho } \right. \\&\quad \left. -2\rho {{\tilde{u}}}\left( \frac{w_{xx}}{\rho } -({{\tilde{u}}}w)_x-ww_x\right) + \frac{\rho _x }{\rho } ({{\tilde{u}}}w)_x \right] \mathrm{d}x. \end{aligned}$$

We now integrate by parts multiple times. Clearly,

$$\begin{aligned} -A^2\int _0^1(\rho w)_x \log \rho \,\mathrm{d}x=A^2\int _0^1 w\rho _x\,\mathrm{d}x=-A^2\int _0^1 w_x\rho \,\mathrm{d}x. \end{aligned}$$

Moreover, expanding all the derivatives and integrating by parts, the second term above reduces to

$$\begin{aligned}&\int _0^1 \left[ \frac{\rho _x (\rho w)_{xx} }{\rho ^3} - \frac{3}{2}\frac{(\rho _x)^2 (\rho w)_x}{\rho ^4}- \frac{\rho _x }{\rho } \left( \frac{w_{xx}}{\rho }-ww_x\right) \right] \mathrm{d}x\\&\quad =\int _0^1 \left[ \frac{1}{2}\frac{\left[ (\rho _x)^2\right] _x w }{\rho ^3}+\frac{1}{2}\frac{(\rho _x)^2w_x }{\rho ^3} - \frac{3}{2}\frac{(\rho _x)^3 w}{\rho ^4}+\frac{\rho _x }{\rho } ww_x\right] \mathrm{d}x\\&\quad =\int _0^1 \frac{\rho _x }{\rho } ww_x\mathrm{d}x. \end{aligned}$$

Concerning the third term, similar arguments lead to

$$\begin{aligned}&\int _0^1 \left[ \left( {{\tilde{u}}}^2 - \frac{\rho _x {{\tilde{u}}}}{\rho ^2}\right) (\rho w)_x +\frac{(\rho w)_{xx} {{\tilde{u}}}}{\rho } \right. \\&\qquad \left. -2\rho {{\tilde{u}}}\left( \frac{w_{xx}}{\rho } -({{\tilde{u}}}w)_x-ww_x\right) + \frac{\rho _x }{\rho } ({{\tilde{u}}}w)_x \right] \mathrm{d}x\\&\quad =\int _0^1 \left[ \left( {{\tilde{u}}}^2 -\left( \frac{{{\tilde{u}}}}{\rho }\right) _x - \frac{\rho _x {{\tilde{u}}}}{\rho ^2}\right) (\rho w)_x \right. \\&\qquad \left. +\,2 {{\tilde{u}}}_x w_{x}+2\rho {{\tilde{u}}}({{\tilde{u}}}w)_x+2\rho {{\tilde{u}}}ww_x + \frac{\rho _x }{\rho } ({{\tilde{u}}}w)_x \right] \mathrm{d}x \\&\quad =\int _0^1 \left[ {{\tilde{u}}}_x w_x+ 3\rho {{\tilde{u}}}^2w_x+{{\tilde{u}}}^2\rho _xw+2\rho {{\tilde{u}}}{{\tilde{u}}}_xw+2\rho {{\tilde{u}}}w w_x+\frac{\rho _x{{\tilde{u}}}}{\rho }w_x\right] \mathrm{d}x\\&\quad =\int _0^1 \left[ {{\tilde{u}}}_x w_x+ 2\rho {{\tilde{u}}}^2w_x+2\rho {{\tilde{u}}}w w_x+\frac{\rho _x{{\tilde{u}}}}{\rho }w_x\right] \mathrm{d}x. \end{aligned}$$

Therefore, collecting all of the above we find that

$$\begin{aligned} {\mathfrak {F}}(\rho ,{{\tilde{u}}}, f,g)&=-A^2\int _0^1 w_x\rho \,\mathrm{d}x-\frac{1}{2} \int _0^1 \frac{\rho _x }{\rho } ww_x\mathrm{d}x\\&\quad -\frac{1}{2}\int _0^1 \left[ {{\tilde{u}}}_x w_x+ 2\rho {{\tilde{u}}}^2w_x+2\rho {{\tilde{u}}}w w_x+\frac{\rho _x{{\tilde{u}}}}{\rho }w_x\right] \mathrm{d}x \end{aligned}$$

and using (1.4), (2.22), (2.6) and standard inequalities we deduce that

$$\begin{aligned}&|{\mathfrak {F}}(\rho ,{{\tilde{u}}}, f,g)|\\&\quad \le A^2\Vert w_x\Vert _{L^\infty }+\Vert w_x\Vert _{L^\infty }^3+\frac{1}{2}\Vert (\log \rho )_x\Vert _{L^2}\Vert w_x \Vert ^2_{L^2}\\&\qquad +\frac{1}{2}\Vert {{\tilde{u}}}_x\Vert _{L^2} \Vert w_{x}\Vert _{L^2} +c\Vert w_x\Vert _{L^\infty }\mathcal {E}(\rho ,{{\tilde{u}}}) \end{aligned}$$

implying that

$$\begin{aligned}&\frac{\mathrm{d}}{\mathrm{d}t} \mathcal {E}(\rho ,{{\tilde{u}}}) + \frac{1}{4} \Vert {{\tilde{u}}}_x\Vert _{L^2}^2 + \frac{A^2}{4} \Vert (\log \rho )_x\Vert _{L^2}^2 \le c\Vert w_x\Vert _{L^\infty }\mathcal {E}(\rho ,{{\tilde{u}}})+\Vert w_x\Vert _{L^\infty }^3 \\&\quad +A^2\Vert w_x\Vert _{L^\infty }+c\Vert w_{x}\Vert _{L^2}^2\left( 1+\frac{1}{A^2}\Vert w_{x}\Vert _{L^2}^2\right) . \end{aligned}$$

Hence, (2.14) follows from the standard Gronwall lemma, together with the fact that

$$\begin{aligned} \mathcal {E}(\rho ,u)&=\int _0^1 \left( \frac{1}{2} \rho ({{\tilde{u}}}+w)^2 + A^2\rho \log \rho \right) \mathrm{d}x+\frac{1}{2} \int _0^1 \left( \frac{\rho _x ({{\tilde{u}}}+w)}{\rho } + \frac{1}{2} \frac{\rho _x^2}{\rho ^3} \right) \mathrm{d}x\\&\le c \mathcal {E}(\rho ,{{\tilde{u}}})+\int _0^1 \left( \rho w^2 + \frac{\rho _x w}{\rho } \right) \mathrm{d}x \le c\mathcal {E}(\rho ,{{\tilde{u}}})+c\Vert w_x\Vert ^2_{L^2}. \end{aligned}$$

#### $H^1$ Estimates on the Velocity

To prove (2.16), we multiply (2.30) by $u_{xx}$ and integrate by parts, to obtain the identity

2.31 $$\begin{aligned}&\frac{\mathrm{d}}{\mathrm{d}t} \Vert {{\tilde{u}}}_x\Vert _{L^2}^2 +2\int _0^1 \frac{|{{\tilde{u}}}_{xx}|^2}{\rho }\mathrm{d}x= 2 \int _0^1 {{\tilde{u}}}{{\tilde{u}}}_x{{\tilde{u}}}_{xx}\mathrm{d}x\nonumber \\&\quad +2 A^2\int _0^1\frac{\rho _x}{\rho }{{\tilde{u}}}_{xx} \mathrm{d}x-2\int _0^1g(\rho ,{{\tilde{u}}},w){{\tilde{u}}}_{xx} \mathrm{d}x. \end{aligned}$$

Now,

$$\begin{aligned}&2\left| \int _0^1 {{\tilde{u}}}{{\tilde{u}}}_x{{\tilde{u}}}_{xx}\mathrm{d}x\right| \\&\quad \le 2 \left( \int _0^1 \frac{|{{\tilde{u}}}_{xx}|^2}{\rho }\mathrm{d}x\right) ^{1/2}\Vert \rho ^{1/2} {{\tilde{u}}}\Vert _{L^\infty }\Vert {{\tilde{u}}}_x\Vert _{L^2}\\&\quad \le 2 \left( \int _0^1 \frac{|{{\tilde{u}}}_{xx}|^2}{\rho }\mathrm{d}x\right) ^{1/2}\Vert \rho \Vert ^{1/2}_{L^\infty }\Vert {{\tilde{u}}}_x\Vert ^2_{L^2}\\&\quad \le \frac{1}{3}\int _0^1 \frac{|{{\tilde{u}}}_{xx}|^2}{\rho }\mathrm{d}x+c\Vert \rho \Vert _{L^\infty }\Vert {{\tilde{u}}}_x\Vert ^4_{L^2}, \end{aligned}$$

and

$$\begin{aligned} 2A^2\left| \int _0^1{{\tilde{u}}}_{xx} \frac{\rho _x}{\rho } \mathrm{d}x\right|&\le \frac{1}{3}\int _0^1 \frac{|{{\tilde{u}}}_{xx}|^2}{\rho }\mathrm{d}x +cA^4 \int _0^1\frac{\rho _x^2}{\rho } \mathrm{d}x\\&\le \frac{1}{3}\int _0^1 \frac{|{{\tilde{u}}}_{xx}|^2}{\rho }\mathrm{d}x +cA^4\Vert \rho \Vert _{L^\infty } \Vert (\log \rho )_x\Vert _{L^2}^2. \end{aligned}$$

Concerning the last term, we find

$$\begin{aligned}&2\left| \int _0^1g(\rho ,{{\tilde{u}}},w){{\tilde{u}}}_{xx} \mathrm{d}x\right| \\&\quad =2\left| \int _0^1\rho ^{1/2}\left( \frac{w_{xx}}{\rho } -({{\tilde{u}}}w)_x-ww_x\right) \frac{{{\tilde{u}}}_{xx}}{\rho ^{1/2}} \mathrm{d}x\right| \\&\quad \le c\left[ \Vert \rho ^{-1}\Vert _{L^\infty }\Vert w_{xx}\Vert ^2_{L^2} +(1+\Vert \rho \Vert _{L^\infty }) \Vert w_x\Vert ^2_{L^\infty }\Vert {{\tilde{u}}}_x\Vert ^2_{L^2} +\Vert w_x\Vert ^4_{L^\infty }\right] \\&\qquad +\frac{1}{3}\int _0^1 \frac{|{{\tilde{u}}}_{xx}|^2}{\rho }\mathrm{d}x. \end{aligned}$$

Thus, (2.31) and the above estimates entail

$$\begin{aligned}&\frac{\mathrm{d}}{\mathrm{d}t} \Vert {{\tilde{u}}}_x\Vert _{L^2}^2 +\int _0^1 \frac{|{{\tilde{u}}}_{xx}|^2}{\rho }\mathrm{d}x\\&\quad \le c\left[ \Vert \rho \Vert _{L^\infty }\Vert {{\tilde{u}}}_x\Vert ^2_{L^2} +(1+\Vert \rho \Vert _{L^\infty }) \Vert w_x\Vert ^2_{L^\infty }\right] \Vert {{\tilde{u}}}_x\Vert ^2_{L^2}\\&\qquad +c\left[ \Vert \rho ^{-1}\Vert _{L^\infty }\Vert w_{xx}\Vert ^2_{L^2} +\Vert w_x\Vert ^4_{L^\infty }+A^4\Vert \rho \Vert _{L^\infty } \Vert (\log \rho )_x\Vert _{L^2}^2\right] . \end{aligned}$$

In view of (2.14), (2.15) and Lemma 2.2, we can apply the Gronwall lemma to the above inequality and deduce (2.16).

### Uniqueness of Strong Solutions

Let $(\rho ,u)$ and (*r*, *v*) be two solutions to (1.1)–(1.2). The relative entropy between $(\rho ,u)$ and (*r*, *v*) is defined as the functional

$$\begin{aligned} \mathcal {H}_{r}(\rho , u | r, v)&= \int _0^1 \left( \frac{1}{2}\rho (u - v)^2 + A^2\rho \log \frac{\rho }{r}\right) \mathrm{d}x. \end{aligned}$$

It is well-known that the above functional is not a distance, and it is not even symmetric. Nonetheless $\mathcal {H}_{r}(\rho , u | r, v)=0$ if and only if $(\rho ,u)=(r,v)$. More precisely, the following well-known facts hold.

#### Lemma 2.5

Let $(\rho ,u)\in \mathcal {X}$ and $(r,v)\in \mathcal {X}$. Then

2.32 $$\begin{aligned} \frac{\min \{1,A^2\}}{2}\min \left\{ \Vert \rho ^{-1}\Vert _{L^\infty } ,\Vert r^{-1}\Vert _{L^\infty } \right\} \left[ \Vert u-v\Vert _{L^2}^2+ \Vert \rho -r\Vert _{L^2}^2\right] \le \mathcal {H}_{r}(\rho , u | r, v) \end{aligned}$$

and

2.33 $$\begin{aligned} \mathcal {H}_{r}(\rho , u | r, v) \le \frac{1}{2} \Vert \rho \Vert _{L^\infty } \Vert u-v\Vert _{L^2}^2 +\frac{A^2}{2}\max \left\{ \Vert \rho ^{-1}\Vert _{L^\infty } , \Vert r^{-1}\Vert _{L^\infty } \right\} \Vert \rho -r\Vert _{L^2}^2. \end{aligned}$$

#### Proof

The proof is based on the fact that the function $\xi \mapsto F(\xi ):=\xi \log \xi -\xi +1$ is convex. In particular,

$$\begin{aligned} F'(\xi )= \log \xi , \qquad F''(\xi )= \frac{1}{\xi }, \qquad \forall \xi >0. \end{aligned}$$

Moreover, due to (1.4), the relative entropy can be rewritten as

$$\begin{aligned} \mathcal {H}_{r}(\rho , u | r, v)&= \int _0^1 \left( \frac{1}{2}\rho (u - v)^2 + A^2\left[ F(\rho )-F(r)-(\rho -r)F'(r)\right] \right) \mathrm{d}x. \end{aligned}$$

Therefore, using Taylor’s theorem, on the one hand we have the lower bound

$$\begin{aligned} \frac{1}{2} \min \left\{ \frac{1}{\xi _1},\frac{1}{\xi _2}\right\} (\xi _1-\xi _2)^2 \le F(\xi _1)-F(\xi _2)-(\xi _1-\xi _2)F'(\xi _2) \end{aligned}$$

while on the other hand the upper bound reads

$$\begin{aligned} F(\xi _1)-F(\xi _2)-(\xi _1-\xi _2)F'(\xi _2) \le \frac{1}{2}\max \left\{ \frac{1}{\xi _1},\frac{1}{\xi _2}\right\} (\xi _1-\xi _2)^2. \end{aligned}$$

Using the above bounds in the expression for $\mathcal {H}_{r}$ yields precisely (2.32)–(2.33), and the proof is over. $\square$

We then have the following uniqueness result.

#### Theorem 2.6

Let $(\rho ,u)$ and (*r*, *v*) be two solutions of (1.1)–(1.5), corresponding to the same initial condition $(\rho _0,u_0)\in \mathcal {X}$. Then

$$\begin{aligned} {\mathbb {P}}\big [(\rho (t),u(t)) =(r(t),v(t)), \ \forall t\ge 0 \big ]=1. \end{aligned}$$

#### Proof

We compute the time derivative of $\mathcal {H}_{r}(\rho , u | r, v)$. Firstly, notice that (1.2) can be rewritten as

$$\begin{aligned} \mathrm{d}u + \left( u u_x + A^2\frac{\rho _x}{\rho } \right) \mathrm{d}t = \frac{u_{xx}}{\rho } \mathrm{d}t + \sigma \mathrm{d}W, \end{aligned}$$

so that the difference of the velocities satisfies

2.34 $$\begin{aligned} (u-v)_t + u u_x -vv_x+ A^2\left[ \log \left( \frac{\rho }{r}\right) \right] _x = \frac{u_{xx}}{\rho }-\frac{v_{xx}}{r} , \end{aligned}$$

Therefore, making use of (1.1) and (2.34), we have that

2.35 $$\begin{aligned}&\frac{\mathrm{d}}{\mathrm{d}t} \frac{1}{2}\int _0^1 \rho (u - v)^2\mathrm{d}x\nonumber \\&\quad = \frac{1}{2}\int _0^1 \rho _t (u - v)^2\mathrm{d}x +\int _0^1 \rho (u - v)(u - v)_t\mathrm{d}x\nonumber \\&\quad =-\frac{1}{2}\int _0^1 (\rho u)_x(u - v)^2\mathrm{d}x+\int _0^1 \rho (u - v)\left( \frac{u_{xx}}{\rho }-\frac{v_{xx}}{r}\right) \mathrm{d}x \nonumber \\&\qquad -\int _0^1 \rho (u - v)(u u_x -vv_x)\mathrm{d}x-A^2\int _0^1 \rho (u - v) \left[ \log \left( \frac{\rho }{r}\right) \right] _x\mathrm{d}x\nonumber \\&\quad =\int _0^1 \rho u(u - v)(u-v)_x\mathrm{d}x- \Vert (u - v)_x\Vert _{L^2}^2 +\int _0^1 \frac{\rho -r}{r} (v -u) v_{xx} \mathrm{d}x\nonumber \\&\qquad -\int _0^1 \rho (u - v)(u u_x -vv_x)\mathrm{d}x-A^2\int _0^1 \rho (u - v) \left[ \log \left( \frac{\rho }{r}\right) \right] _x\mathrm{d}x\nonumber \\&\quad =-\Vert (u - v)_x\Vert _{L^2}^2+\int _0^1 \frac{\rho -r}{r} (v -u) v_{xx} \mathrm{d}x- \int _0^1 v_x \rho (u - v)^2 \mathrm{d}x\nonumber \\&\qquad -A^2\int _0^1 \rho (u - v) \left[ \log \left( \frac{\rho }{r}\right) \right] _x\mathrm{d}x. \end{aligned}$$

On the other hand, due to (1.1) once more, we compute

2.36 $$\begin{aligned} \frac{\mathrm{d}}{\mathrm{d}t} \int _0^1 \rho \log \left( \frac{\rho }{r}\right) \mathrm{d}x&=\int _0^1 \rho _t\log \left( \frac{\rho }{r}\right) \mathrm{d}x +\int _0^1 \left( \rho _t- \frac{\rho }{r}r_t\right) \mathrm{d}x\nonumber \\&=-\int _0^1 (\rho u)_x\log \left( \frac{\rho }{r}\right) \mathrm{d}x-\int _0^1 (\rho u)_x\mathrm{d}x +\int _0^1\frac{\rho }{r}(r v)_x\mathrm{d}x\nonumber \\&=\int _0^1 \rho u\left[ \log \left( \frac{\rho }{r}\right) \right] _x\mathrm{d}x+\int _0^1\rho v_x\mathrm{d}x+\int _0^1\frac{\rho }{r}r_x v\mathrm{d}x\nonumber \\&=\int _0^1 \rho u\left[ \log \left( \frac{\rho }{r}\right) \right] _x\mathrm{d}x-\int _0^1\rho _x v\mathrm{d}x+\int _0^1\frac{\rho }{r}r_x v\mathrm{d}x\nonumber \\&=\int _0^1 \rho (u - v) \left[ \log \left( \frac{\rho }{r}\right) \right] _x. \end{aligned}$$

Therefore, combining (2.35) and (2.36), we arrive at

2.37 $$\begin{aligned} \frac{\mathrm{d}}{\mathrm{d}t}\mathcal {H}_{r}(\rho , u | r, v) +\Vert (u - v)_x\Vert _{L^2}^2&=\int _0^1 \frac{\rho -r}{r} (v -u) v_{xx} \mathrm{d}x- \int _0^1 v_x \rho (u - v)^2 \mathrm{d}x.\end{aligned}$$

We now estimate each term in the right-hand side above. We preliminary notice that for any smooth function *g* such that $g(x_0)=0$ for some $x_0\in [0,1]$, there holds the inequality

2.38 $$\begin{aligned} \Vert g\Vert _{L^\infty } \le \left( \int _0^1 \frac{|g_x|^2}{\rho }\mathrm{d}x\right) ^{1/2}, \end{aligned}$$

where $\rho$ can be replaced by any positive function with mass at most 1 (and, in particular, by *r*). Indeed,

$$\begin{aligned} |g(x)|&=\left| \int _{x_0}^x g_y\mathrm{d}y\right| =\left| \int _{x_0}^x \rho ^{1/2}\frac{g_y}{\rho ^{1/2}}\mathrm{d}y\right| \\&\le \left( \int _{x_0}^x \rho \mathrm{d}y\right) ^{1/2} \left( \int _{x_0}^x\frac{|g_y|^2}{\rho }\mathrm{d}y\right) ^{1/2}\le \left( \int _0^1 \frac{|g_x|^2}{\rho }\mathrm{d}x\right) ^{1/2}. \end{aligned}$$

Concerning the first term in the right-hand side of (2.37), using standard inequalities and (2.32) we obtain

$$\begin{aligned}&\int _0^1 \frac{\rho -r}{r} (v -u) v_{xx} \mathrm{d}x \\&\quad \le \Vert r^{-1}\Vert ^{1/2}_{L^\infty }\Vert \rho -r\Vert _{L^2}\Vert v-u\Vert _{L^\infty } \left( \int _0^1\frac{|v_{xx}|^2}{r}\mathrm{d}x\right) ^{1/2} \\&\quad \le \Vert r^{-1}\Vert _{L^\infty }\left( \int _0^1\frac{|v_{xx}|^2}{r}\mathrm{d}x\right) \Vert \rho -r\Vert _{L^2}^2 +\frac{1}{2}\Vert (u-v)_x\Vert _{L^2}^2\\&\quad \le \frac{2}{\min \{1,A^2\}}\frac{\Vert r^{-1}\Vert _{L^\infty } }{\min \left\{ \Vert \rho ^{-1}\Vert _{L^\infty } ,\Vert r^{-1}\Vert _{L^\infty } \right\} }\left( \int _0^1\frac{|v_{xx}|^2}{r}\mathrm{d}x\right) \mathcal {H}_{r}(\rho , u | r, v)\\&\qquad +\frac{1}{2}\Vert (u-v)_x\Vert _{L^2}^2\\&\quad \le \frac{2}{\min \{1,A^2\}}\max \left\{ 1, \frac{\Vert r^{-1}\Vert _{L^\infty } }{\Vert \rho ^{-1}\Vert _{L^\infty } }\right\} \left( \int _0^1\frac{|v_{xx}|^2}{r}\mathrm{d}x\right) \mathcal {H}_{r}(\rho , u | r, v) \\&\qquad +\frac{1}{2}\Vert (u-v)_x\Vert _{L^2}^2, \end{aligned}$$

where the last line follows from (2.32). For the second term, we use (2.22) and (2.38) to deduce that

$$\begin{aligned} \int _0^1 v_x \rho (u - v)^2 \mathrm{d}x&\le \Vert v_x\Vert _{L^\infty }\left( \int _0^1 \rho (u - v)^2 \mathrm{d}x\right) ^{1/2}\Vert (u-v)_x\Vert _{L^2}\\&\le \left( \int _0^1\frac{|v_{xx}|^2}{r}\mathrm{d}x\right) \mathcal {H}_{r}(\rho , u | r, v)+\frac{1}{2}\Vert (u-v)_x\Vert _{L^2}^2. \end{aligned}$$

Hence, from (2.37) it follows that

2.39 $$\begin{aligned}&\frac{\mathrm{d}}{\mathrm{d}t}\mathcal {H}_{r}(\rho , u | r, v) \nonumber \\&\quad \le \left( 1+\frac{2}{\min \{1,A^2\}}\max \left\{ 1, \frac{\Vert r^{-1}\Vert _{L^\infty } }{\Vert \rho ^{-1}\Vert _{L^\infty } }\right\} \right) \left( \int _0^1\frac{|v_{xx}|^2}{r}\mathrm{d}x\right) \mathcal {H}_{r}(\rho , u | r, v). \end{aligned}$$

Thanks to Proposition 2.4, the term multiplying $\mathcal {H}_{r}(\rho , u | r, v)$ in the right-hand side above is locally integrable in time. We now apply the standard Gronwall lemma to (2.39) and use the fact that

$$\begin{aligned} \mathcal {H}_{r}(\rho , u | r, v)|_{t=0}=0 \end{aligned}$$

by assumption to conclude that the two solutions are indistinguishable. $\square$

### Continuous Dependence of Solutions

Regarding the continuous dependence of solutions, the picture is more complicated, and can be proven in the following situation.

#### Theorem 2.7

Let $\{(\rho ^n,u^n)\}_{n\in {\mathbb {N}}}$ be a sequence of solutions to (1.1)–(1.5) with initial data $\{(\rho _0^n,u_0^n)\}_{n\in {\mathbb {N}}}$ such that

2.40 $$\begin{aligned} \sup _{n\in {\mathbb {N}}}\mathcal {E}(\rho _0^n,u_0^n)<\infty , \end{aligned}$$

and let $(\rho ,u)$ be a a solution to (1.1)–(1.5) with initial datum $(\rho _0,u_0)\in \mathcal {X}$. If

2.41 $$\begin{aligned} (\rho _0^n,u_0^n)\rightarrow (\rho _0,u_0) \quad \text {in } L^2\times L^2, \end{aligned}$$

then

$$\begin{aligned} (\rho ^n,u^n)\rightarrow (\rho ,u) \quad \text {a.s. in } L^2\times L^2, \end{aligned}$$

for all $$t\ge 0$$.

#### Proof

We use the approach in Theorem 2.6. Arguing in the same way as we did to derive (2.39), we arrive at

2.42 $$\begin{aligned}&\frac{\mathrm{d}}{\mathrm{d}t}\mathcal {H}_{r}(\rho ^n, u^n | \rho , u) \nonumber \\&\quad \le \left( 1+\frac{2}{\min \{1,A^2\}}\max \left\{ 1, \frac{\Vert \rho ^{-1}\Vert _{L^\infty } }{\Vert (\rho ^n)^{-1}\Vert _{L^\infty } } \right\} \right) \left( \int _0^1\frac{|u_{xx}|^2}{\rho }\mathrm{d}x\right) \mathcal {H}_{r}(\rho ^n, u^n | \rho , u). \end{aligned}$$

Due to the lack of symmetry of the relative entropy, it is important that we consider the above quantity, and not the other possible $\mathcal {H}_{r}(\rho , u | \rho ^n, u^n)$. Let

$$\begin{aligned} R=\max \left\{ \sup _{n\in {\mathbb {N}}}\mathcal {E}(\rho _0^n,u_0^n), \mathcal {E}(\rho _0,u_0)\right\} <\infty . \end{aligned}$$

In view of Proposition 2.4, we have *n*-independent almost sure bounds of the form

$$\begin{aligned} \sup _{t\in [0,T]}\int _0^t\left( \int _0^1\frac{|u_{xx}|^2}{\rho }\mathrm{d}x\right) \mathrm{d}s\le C\left( T,\sigma W, R, \Vert (u_0)_x\Vert _{L^2}\right) , \end{aligned}$$

and

$$\begin{aligned} \sup _{t\in [0,T]}\frac{\Vert \rho ^{-1}\Vert ^2_{L^\infty } }{\Vert \rho _n^{-1}\Vert ^2_{L^\infty }}\le C\left( T,\sigma W,R\right) . \end{aligned}$$

The point is that the latter bound does not depend on $\Vert (u^n_0)_x\Vert _{L^2}$, which is not, in general, under control uniformly in $n\in {\mathbb {N}}$. Thanks to Lemma 2.5, the fact that $\mathcal {E}(\rho ^n_0, u_0^n)\le R$ and (2.41), we have that

$$\begin{aligned} \lim _{n\rightarrow \infty } \mathcal {H}_{r}(\rho _0^n, u_0^n | \rho _0, u_0)=0. \end{aligned}$$

Thus, the standard Gronwall lemma applied to (2.42) implies

$$\begin{aligned} \lim _{n\rightarrow \infty }\mathcal {H}_{r}(\rho ^n(t), u^n(t) | \rho (t), u(t))=0, \qquad \text {a.s.} \end{aligned}$$

for all $t\ge 0$. Hence, a further application of Lemma 2.5 leads to the conclusion of the proof. $\square$

## Invariant Measures

In this section, we prove of the main results of this article, namely the existence of invariant measures to (1.1)–(1.2). As mentioned earlier, we will work in the phase space

$$\begin{aligned} \mathcal {X}=\left\{ (\rho ,u)\in H^1(0,1)\times H^1_0(0,1): \int _0^1 \rho (x) \mathrm{d}x = 1, \ \rho >0\right\} . \end{aligned}$$

However, in view of Theorem 2.7, the correct topology does not seem to be the natural one induced by the product norm in $\mathcal {X}$, since Lemma 2.5 suggests that continuous dependence for our system holds in a weaker sense. We make this precise here below.

### The Markovian Framework

We denote by $\rho (t;\rho _0), u(t;u_0)$ the unique solution to (1.1)–(1.2), with (deterministic) initial data $(\rho _0, u_0)\in \mathcal {X}$. For a set $B\in \mathcal {B}(\mathcal {X}_{L^2})$, we define the transition functions

$$\begin{aligned} {\mathcal {P}}_t(\rho _0, u_0, B)={\mathbb {P}}((\rho (t;\rho _0), u(t;u_0)) \in B), \end{aligned}$$

for any $t\ge 0$. For $t\ge 0$, define the Markov semigroup

$$\begin{aligned} {\mathcal {P}}_t \phi (\rho _0, u_0)= {\mathbb {E}}\phi (\rho (t;\rho _0), u(t;u_0)), \qquad \phi \in M_b(\mathcal {X}_{L^2}). \end{aligned}$$

The usual semigroup properties

$$\begin{aligned} {\mathcal {P}}_0=\text {identity on }M_b(\mathcal {X}_{L^2}) \end{aligned}$$

and

$$\begin{aligned} {\mathcal {P}}_{t+s}={\mathcal {P}}_t\circ {\mathcal {P}}_s, \qquad \forall t,s\ge 0, \end{aligned}$$

follow from the existence and uniqueness results for our system. The goal is to prove the existence of a probability measure $\mu \in {\mathfrak {P}}(\mathcal {X}_{L^2})$ that is *invariant* under ${\mathcal {P}}_t$, namely such that

$$\begin{aligned} \int _\mathcal {X}{\mathcal {P}}_t\phi \, \mathrm{d}\mu = \int _\mathcal {X}\phi \, \mathrm{d}\mu , \qquad \forall \phi \in C_b(\mathcal {X}_{L^2}). \end{aligned}$$

Now, it is clear from the estimates in Propositions 2.3 and 2.4 that

$$\begin{aligned} {\mathcal {P}}_t:M_b(\mathcal {X}_{L^2})\rightarrow M_b(\mathcal {X}_{L^2}), \end{aligned}$$

namely, ${\mathcal {P}}_t$ is well defined on measurable bounded functions. The Feller property, namely that ${\mathcal {P}}_t$ maps $C_b(\mathcal {X}_{L^2})$ into $C_b(\mathcal {X}_{L^2})$ is more delicate, and we are not able to prove it at the moment. In particular, it does not follow from Theorem 2.7, due to the additional requirement (2.40). However, Theorem 2.7 suggests the following definition.

#### Definition 3.1

Let $\phi \in M_b(\mathcal {X}_{L^2})$. We say that $\phi :\mathcal {X}\rightarrow {\mathbb {R}}$ belongs to the class $\mathcal {G}$ if

$$\begin{aligned} \lim _{n\rightarrow \infty }\phi (\rho ^n,u^n)=\phi (\rho ,u), \end{aligned}$$

whenever

3.1 $$\begin{aligned} \{(\rho ^n,u^n)\}_{n\in {\mathbb {N}}}\subset \mathcal {X}_{L^2}, \quad \lim _{n\rightarrow \infty }(\rho ^n,u^n)=(\rho ,u) \quad \text {in } \mathcal {X}_{L^2}, \quad \text {and}\quad \sup _{n\in {\mathbb {N}}}\mathcal {E}(\rho ^n,u^n)<\infty . \end{aligned}$$

From the above definition and Lemma 2.2, it is clear that

$$\begin{aligned} C_b(\mathcal {X}_{L^2})\subset \mathcal {G}\subset C_b(\mathcal {X}_{H^1}). \end{aligned}$$

It turns out that $\mathcal {G}$ is also invariant under ${\mathcal {P}}_t$.

#### Lemma 3.2

The class $\mathcal {G}$ is invariant under the Markov semigroup ${\mathcal {P}}_t$, namely

$$\begin{aligned} {\mathcal {P}}_t:\mathcal {G}\rightarrow \mathcal {G}. \end{aligned}$$

#### Proof

Let $\{(\rho _0^n,u_0^n)\}_{n\in {\mathbb {N}}}\subset \mathcal {X}_{L^2}$ be a sequence, complying with (3.1), namely

3.2 $$\begin{aligned} \lim _{n\rightarrow \infty }(\rho _0^n,u_0^n)=(\rho _0,u_0) \quad \text {in } \mathcal {X}_{L^2}, \quad \text {and}\quad M:=\sup _{n\in {\mathbb {N}}}\mathcal {E}(\rho _0^n,u_0^n)<\infty . \end{aligned}$$

In light of Theorem 2.7,

3.3 $$\begin{aligned} \lim _{n\rightarrow \infty }(\rho (t;\rho _0^n),u(t;u_0^n))=(\rho (t;\rho _0),u(t;u_0)) \quad \text {a.s. in } \mathcal {X}_{L^2},\quad \forall t\ge 0. \end{aligned}$$

Moreover, in view of (2.10) and (3.2), there exists a constant ${\bar{M}}:={\bar{M}}(t,M,\sigma )$ independent of $n\in {\mathbb {N}}$ such that

$$\begin{aligned} {\mathbb {E}}\mathcal {E}(\rho (t;\rho ^n_0), u(t;u^n_0))\le {\bar{M}}, \end{aligned}$$

which, by Chebyshev’s inequality, it implies that

3.4 $$\begin{aligned} {\mathbb {P}}\left[ \mathcal {E}(\rho (t;\rho ^n_0), u(t;u^n_0)) > R\right] \le \frac{{\bar{M}}}{R}, \end{aligned}$$

for any $R>0$. Fix $\phi \in \mathcal {G}$, and let

3.5 $$\begin{aligned} M_\phi :=\sup _{(\rho ,u)\in \mathcal {X}_{L^2}} |\phi (\rho ,u)|<\infty . \end{aligned}$$

To show that ${\mathcal {P}}_t \phi \in \mathcal {G}$, we are required to prove that for any fixed $t>0$ there holds

$$\begin{aligned} \lim _{n\rightarrow \infty }{\mathcal {P}}_t \phi (\rho ^n_0, u^n_0)= {\mathcal {P}}_t \phi (\rho _0, u_0). \end{aligned}$$

Writing $\mathcal {E}_n=\mathcal {E}(\rho (t;\rho ^n_0), u(t;u^n_0))$ for short, we have by definition that

$$\begin{aligned}&{\mathcal {P}}_t \phi (\rho ^n_0, u^n_0)-{\mathcal {P}}_t \phi (\rho _0, u_0)\\&\quad ={\mathbb {E}}\left[ \phi (\rho (t;\rho ^n_0), u(t;u^n_0))-\phi (\rho (t;\rho _0), u(t;u_0))\right] \\&\quad ={\mathbb {E}}\left[ (\phi (\rho (t;\rho ^n_0), u(t;u^n_0))-\phi (\rho (t;\rho _0), u(t;u_0)))1 \! \! 1_{\mathcal {E}_n\le R}\right] \\&\qquad +{\mathbb {E}}\left[ (\phi (\rho (t;\rho ^n_0), u(t;u^n_0))-\phi (\rho (t;\rho _0), u(t;u_0)))1 \! \! 1_{\mathcal {E}_n> R}\right] . \end{aligned}$$

In light of (3.4)–(3.5),

3.6 $$\begin{aligned} \left| {\mathbb {E}}\left[ (\phi (\rho (t;\rho ^n_0), u(t;u^n_0))-\phi (\rho (t;\rho _0), u(t;u_0)))1 \! \! 1_{\mathcal {E}_n> R}\right] \right| \le \frac{2 {\bar{M}}M_\phi }{R}. \end{aligned}$$

Since $\phi \in \mathcal {G}$, we also deduce by (3.2) and (3.3) that

$$\begin{aligned} \lim _{n\rightarrow \infty } (\phi (\rho (t;\rho ^n_0), u(t;u^n_0))-\phi (\rho (t;\rho _0), u(t;u_0)))1 \! \! 1_{\mathcal {E}_n\le R}=0, \qquad \text {a.s.}, \end{aligned}$$

and therefore by the bounded convergence theorem that

3.7 $$\begin{aligned} \lim _{n\rightarrow \infty }{\mathbb {E}}\left[ (\phi (\rho (t;\rho ^n_0), u(t;u^n_0))-\phi (\rho (t;\rho _0), u(t;u_0)))1 \! \! 1_{\mathcal {E}_n\le R}\right] =0. \end{aligned}$$

Let us now arbitrarily fix $\varepsilon >0$, and pick $R_\varepsilon =4{\bar{M}}M_\phi /\varepsilon$. Invoking (3.6), we deduce that

$$\begin{aligned} {\mathbb {E}}\left[ (\phi (\rho (t;\rho ^n_0), u(t;u^n_0))-\phi (\rho (t;\rho _0), u(t;u_0)))1 \! \! 1_{\mathcal {E}_n> R}\right] \le \frac{\varepsilon }{2}. \end{aligned}$$

Moreover, from (3.7) there exists $n_\varepsilon \in {\mathbb {N}}$ such that

$$\begin{aligned} \left| {\mathbb {E}}\left[ (\phi (\rho (t;\rho ^n_0), u(t;u^n_0))-\phi (\rho (t;\rho _0), u(t;u_0)))1 \! \! 1_{\mathcal {E}_n\le R}\right] \right| <\frac{\varepsilon }{2}, \qquad \forall n\ge n_\varepsilon . \end{aligned}$$

Thus,

$$\begin{aligned} |{\mathcal {P}}_t \phi (\rho ^n_0, u^n_0)-{\mathcal {P}}_t \phi (\rho _0, u_0)|< \varepsilon , \qquad \forall t\ge 0, n\ge n_\varepsilon , \end{aligned}$$

and the proof is over. $\square$

### Tightness of Time-Averaged Measures

We prove the existence of an invariant measure for ${\mathcal {P}}_t$ via the classical Krylov–Bogoliubov procedure. However, since we are working in a non-complete metric space, and with a Markov semigroup that is not Feller, some details do not follow directly from the well-known theory. In what follows, the parameter $A>0$ appearing in (1.2) is fixed, but we suppress the dependence on it of all the quantities (except for bounds), in order to keep the notation as simple as possible. As initial conditions to our problem, we fix $\rho _0=1$ and $u_0=0$. Notice that the corresponding energy vanishes, namely $\mathcal {E}(1, 0)=0$. For $T>0$, define the time-averaged measure on $\mathcal {X}$ by

3.8 $$\begin{aligned} \mu _T(B)=\frac{1}{T}\int _0^T{\mathbb {P}}(\rho (t;1),u(t;0)\in B) \mathrm{d}t, \end{aligned}$$

where $B\in \mathcal {B}(\mathcal {X}_{L^2})$. The first step is to prove tightness of the family $\{\mu _T\}_{T>0}$. We will make use of the following lemma.

#### Lemma 3.3

Assume that $\rho \in H^1$ is such that $\rho > 0$ and

$$\begin{aligned} \int _0^1\rho \, \mathrm{d}x=1, \qquad M^2:=\int _0^1 \frac{\rho _x^2}{\rho ^2}\mathrm{d}x=\int _0^1 [(\log \rho )_x]^2\mathrm{d}x <\infty . \end{aligned}$$

Then, for $x\in [0,1]$, we have the pointwise bounds

3.9 $$\begin{aligned} \mathrm{e}^{-M}\le \rho (x)\le \mathrm{e}^M, \end{aligned}$$

and, moreover

$$\begin{aligned} \int _0^1 \rho _x^2 \mathrm{d}x\le M^2\mathrm{e}^{2M}. \end{aligned}$$

#### Proof

Firstly, we notice since $\rho$ is continuous and has mean 1, there exists $x_0\in (0,1)$ such that $\rho (x_0)=1$. Therefore, for every $x\in [0,1]$ we have

$$\begin{aligned} |\log \rho (x)|=\left| \int _{x_0}^x (\log \rho )_y \mathrm{d}y \right| \le \left[ \int _0^1[(\log \rho )_y]^2\mathrm{d}y\right] ^{1/2}=M, \qquad \forall x\in [0,1]. \end{aligned}$$

As a consequence,

$$\begin{aligned} \mathrm{e}^{-M}\le \rho (x)\le \mathrm{e}^{M}, \qquad \forall x\in [0,1], \end{aligned}$$

proving (3.9). Moreover,

$$\begin{aligned} \int _0^1 \rho _x^2\mathrm{d}x\le \Vert \rho \Vert _{L^\infty }^2\int _0^1\frac{\rho _x^2}{\rho ^{2}}\mathrm{d}x. \end{aligned}$$

The claim then follows from (3.9). $\square$

As a consequence, tightness of $\{\mu _T\}_{T>0}$ follows in a straightforward manner.

#### Proposition 3.4

The family of probability measures $\{\mu _T\}_{T>0}\subset {\mathfrak {P}}(\mathcal {X}_{L^2})$ is tight. Hence, there exists a subsequence $T_j\rightarrow \infty$ and a measure $\mu \in {\mathfrak {P}}(\mathcal {X}_{L^2})$ such that

3.10 $$\begin{aligned} \lim _{j\rightarrow \infty } \int _\mathcal {X}\phi \, \mathrm{d}\mu _{T_j} = \int _\mathcal {X}\phi \, \mathrm{d}\mu , \qquad \forall \phi \in C_b(\mathcal {X}_{L^2}). \end{aligned}$$

#### Proof

For any fixed $R,S\ge 1$, define the sets

$$\begin{aligned} K_R=\left\{ (\rho ,u)\in \mathcal {X}: \int _0^1 u_x^2 \mathrm{d}x+ \int _0^1 \frac{\rho _x^2}{\rho ^{2}}\mathrm{d}x \le R^2\right\} \end{aligned}$$

and

3.11 $$\begin{aligned} C_S=\left\{ (\rho ,u)\in \mathcal {X}: \int _0^1 u_x^2 \mathrm{d}x + \int _0^1\rho _x^2\mathrm{d}x+ \Vert \rho \Vert _{L^\infty }+\Vert \rho ^{-1}\Vert _{L^\infty }\le S\right\} . \end{aligned}$$

Note that since $C_S$ is bounded in $H^1\times H^1_0$ and is closed, it is compact in $\mathcal {X}_{L^2}$. By Lemma 3.3, we have that if $(\rho ,u)\in K_R$, then

$$\begin{aligned} \int _0^1\rho _x^2\mathrm{d}x\le R^2\mathrm{e}^{2R},\quad \int _0^1 u_x^2 \mathrm{d}x \le R^2,\quad \Vert \rho \Vert _{L^\infty }\le \mathrm{e}^{R},\quad \Vert \rho ^{-1}\Vert _{L^\infty }\le \mathrm{e}^R, \end{aligned}$$

In particular, if $R\ge 1$, we obtain

3.12 $$\begin{aligned} \int _0^1\rho _x^2\mathrm{d}x+\int _0^1 u_x^2 \mathrm{d}x + \Vert \rho \Vert _{L^\infty }+\Vert \rho ^{-1}\Vert _{L^\infty }\le S_R:=4R^2\mathrm{e}^{2R}, \end{aligned}$$

which therefore translates into the set inclusion

3.13 $$\begin{aligned} K_R\subset C_{S_R}. \end{aligned}$$

In view of (3.13) and Chebyshev’s inequality we have

$$\begin{aligned} \mu _T(C_{S_R})&\ge \mu _T (K_R)=1-\mu _T (\mathcal {X}\setminus K_R)\\&=1-\frac{1}{T}\int _0^T {\mathbb {P}}\left[ \int _0^1 \frac{\rho _x^2}{\rho ^{2}}\mathrm{d}x+ \int _0^1 u_x^2 \mathrm{d}x > R^2\right] \\&\ge 1-\frac{1}{R^2} \frac{1}{T} \int _0^T {\mathbb {E}}\left[ \int _0^1 \frac{\rho _x^2}{\rho ^{2}}\mathrm{d}x+ \int _0^1 u_x^2 \mathrm{d}x\right] . \end{aligned}$$

In light of the energy inequality (2.10) and the fact that $\mathcal {E}(1, 0)=0$, it follows that

3.14 $$\begin{aligned} \mu _T(C_{S_R})\ge 1-\frac{\Vert \sigma \Vert _{L^\infty }^2}{\min \{1,A^2\}}\frac{1}{R^2}. \end{aligned}$$

Hence, the family $\{\mu _T\}_{T>0}$ is tight, and therefore there exists a subsequential limit $\mu \in {\mathfrak {P}}(\mathcal {X}_{L^2})$. Note that this uses the direction of Prokhorov’s theorem that does not require completeness (see [1, Theorem 6.1]). Now, since $C_{S_R}$ is a closed set, the Portmanteau theorem implies

3.15 $$\begin{aligned} \mu (\mathcal {X})\ge \mu (C_{S_R}) \ge \limsup _{T\rightarrow \infty }\mu _T(C_{S_R}) \ge 1-\frac{\Vert \sigma \Vert _{L^\infty }^2}{\min \{1,A^2\}}\frac{1}{R^2}. \end{aligned}$$

Notice again that this does not require the metric space $\mathcal {X}_{L^2}$ to be complete (see [1, Theorem 2.1]). Thus

$$\begin{aligned} \mu (\mathcal {X})\ge \lim _{R\rightarrow \infty }\mu (C_{S_R})=1. \end{aligned}$$

The proof is therefore concluded. $\square$

### Invariance of the Limit Measure

We aim to prove the following proposition.

#### Proposition 3.5

For every fixed $A>0$, the Markov semigroup $\{{\mathcal {P}}_t\}_{t\ge 0}$ associated to (1.1)–(1.5) possesses an invariant probability measure $\mu _A\in {\mathfrak {P}}(\mathcal {X}_{L^2})$. Furthermore,

3.16 $$\begin{aligned} \int _{\mathcal {X}} \left[ A^2\Vert (\log \rho )_x\Vert _{L^2}^2+\Vert u_x\Vert _{L^2}^2\right] \mu _A(\rho ,u) \le \Vert \sigma \Vert ^2_{L^\infty }. \end{aligned}$$

The main issue here is that ${\mathcal {P}}_t$ is not known to be Feller, and therefore we cannot directly apply (3.10) to ${\mathcal {P}}_t\phi$. As a first step, we extend property (3.10) to the class $\mathcal {G}$.

#### Lemma 3.6

Let $\{\mu _{T_j}\}_{j\in {\mathbb {N}}}\subset {\mathfrak {P}}(\mathcal {X}_{L^2})$ be the convergent sequence in Proposition 3.4. Then

$$\begin{aligned} \lim _{j\rightarrow \infty } \int _\mathcal {X}\phi \, \mathrm{d}\mu _{T_j} = \int _\mathcal {X}\phi \, \mathrm{d}\mu , \qquad \forall \phi \in \mathcal {G}. \end{aligned}$$

#### Proof

Let $\phi \in \mathcal {G}$, arbitrarily fix $R\ge 1$, and consider the compact sets $C_{S_R}$ from (3.11), where $S_R$ is given by (3.12). Since $\phi \in \mathcal {G}$, $\phi$ is bounded on $\mathcal {X}_{L^2}$, so that

$$\begin{aligned} M_\phi :=\sup _{(\rho ,u)\in \mathcal {X}_{L^2}} |\phi (\rho ,u)|<\infty . \end{aligned}$$

Moreover, the restriction of $\phi$ to $C_{S_R}$ is continuous (in the $L^2\times L^2$ metric) on $C_{S_R}$, for every $R\ge 1$. Since $C_{S_R}$ is a closed set, Tietze extension theorem (see e.g. [35]) guarantees the existence of a function ${{\tilde{\phi }}}_R\in C_b(\mathcal {X}_{L^2})$ such that

3.17 $$\begin{aligned} {\tilde{\phi }}_R=\phi \quad \text {on } C_{S_R}, \qquad \sup _{(\rho ,u)\in \mathcal {X}_{L^2}}|{\tilde{\phi }}_R(\rho ,u)|\le M_\phi , \qquad \forall R\ge 1. \end{aligned}$$

Now,

$$\begin{aligned}&\left| \int _{\mathcal {X}}\phi \, \mathrm{d}\mu _{T_j}-\int _\mathcal {X}\phi \, \mathrm{d}\mu \right| \le \left| \int _{\mathcal {X}}(\phi -{\tilde{\phi }}_R)\, \mathrm{d}\mu _{T_j}\right| \\&\quad + \left| \int _{\mathcal {X}}{\tilde{\phi }}_R\, \mathrm{d}\mu _{T_j}-\int _\mathcal {X}{\tilde{\phi }}_R\, \mathrm{d}\mu \right| + \left| \int _{\mathcal {X}}({\tilde{\phi }}_R-\phi )\, \mathrm{d}\mu \right| \end{aligned}$$

Fix $\varepsilon >0$. By (3.14) and the above (3.17), there exists $R_\varepsilon \ge 1$ such that

$$\begin{aligned}&\left| \int _{\mathcal {X}}(\phi -{\tilde{\phi }}_{R_\varepsilon })\, \mathrm{d}\mu _{T_j}\right| =\left| \int _{\mathcal {X}\setminus C_{S_{R_\varepsilon }}}(\phi -{\tilde{\phi }}_{R_\varepsilon })\, \mathrm{d}\mu _{T_j}\right| \le 2M_\phi \mu _{T_j}(\mathcal {X}\setminus C_{S_{R_\varepsilon }})\\&\quad \le \frac{2M_\phi \Vert \sigma \Vert _{L^\infty }^2}{\min \{1,A^2\}}\frac{1}{R_\varepsilon ^2}<\frac{\varepsilon }{3}. \end{aligned}$$

uniformly for $j\in {\mathbb {N}}$, and, similarly, in light of (3.15),

$$\begin{aligned} \left| \int _{\mathcal {X}}({\tilde{\phi }}_{R_\varepsilon }-\phi )\, \mathrm{d}\mu \right| <\frac{\varepsilon }{3}. \end{aligned}$$

Since ${\tilde{\phi }}_{R_\varepsilon }\in C_b(\mathcal {X}_{L^2})$, property (3.10) implies the existence of $j_\varepsilon \in {\mathbb {N}}$ such that

$$\begin{aligned} \left| \int _{\mathcal {X}}{\tilde{\phi }}_{R_\varepsilon }\, \mathrm{d}\mu _{T_j}-\int _\mathcal {X}{\tilde{\phi }}_{R_\varepsilon }\, \mathrm{d}\mu \right| <\frac{\varepsilon }{3},\qquad \forall j\ge j_\varepsilon . \end{aligned}$$

Hence, for every $\varepsilon >0$, there exists $j_\varepsilon \in {\mathbb {N}}$ such that

$$\begin{aligned} \left| \int _{\mathcal {X}}\phi \, \mathrm{d}\mu _{T_j}-\int _\mathcal {X}\phi \, \mathrm{d}\mu \right| <\varepsilon , \qquad \forall j\ge j_\varepsilon , \end{aligned}$$

which proves the claim. $\square$

We are now in the position to prove invariance of the limiting measure and the bound (3.16).

#### Proof of Proposition 3.5

As before, $A>0$ is fixed and we suppress the various dependences on it. Let $\phi \in C_b(\mathcal {X}_{L^2})$ be fixed. Thanks to Lemma 3.2,

$$\begin{aligned} {\mathcal {P}}_t\phi \in \mathcal {G}, \qquad \forall t>0. \end{aligned}$$

In view of Lemma 3.6, we then have that

$$\begin{aligned} \lim _{j\rightarrow \infty } \int _\mathcal {X}{\mathcal {P}}_t\phi \, \mathrm{d}\mu _{T_j} = \int _\mathcal {X}{\mathcal {P}}_t\phi \, \mathrm{d}\mu , \qquad \forall t\ge 0. \end{aligned}$$

Hence, by (3.8), we conclude that

$$\begin{aligned}&\int _\mathcal {X}{\mathcal {P}}_t\phi \, \mathrm{d}\mu \\&\quad =\lim _{j\rightarrow \infty } \int _\mathcal {X}{\mathcal {P}}_t\phi \, \mathrm{d}\mu _{T_j} =\lim _{j\rightarrow \infty } \frac{1}{T_j}\int _0^{T_j} {\mathcal {P}}_{t+s} \phi (1,0)\mathrm{d}s =\lim _{j\rightarrow \infty } \frac{1}{T_j}\int _t^{T_j+t} {\mathcal {P}}_{s} \phi (1,0)\mathrm{d}s\\&\quad =\lim _{j\rightarrow \infty } \left( \frac{1}{T_j}\int _0^{T_j} {\mathcal {P}}_{s} \phi (1,0)\mathrm{d}s+ \frac{1}{T_j}\int _{T_j}^{T_j+t} {\mathcal {P}}_{s} \phi (1,0)\mathrm{d}s -\frac{1}{T_j}\int _0^{t} {\mathcal {P}}_{s} \phi (1,0)\mathrm{d}s\right) \\&\quad =\lim _{j\rightarrow \infty } \int _\mathcal {X}\phi \, \mathrm{d}\mu _{T_j} = \int _\mathcal {X}\phi \, \mathrm{d}\mu , \end{aligned}$$

so that invariance follows from the arbitrariness of $\phi$. Lastly, (3.16) is deduced directly from (2.10). Indeed, is $(\rho ^S,u^S)\in \mathcal {X}$ is a statistically stationary solution distributed as $\mu$, the we can use (2.10) to derive the bound

$$\begin{aligned} {\mathbb {E}}\Vert u^S_x\Vert ^2_{L^2} + A^2{\mathbb {E}}\Vert (\log \rho ^S)_x\Vert _{L^2}^2 \le \Vert \sigma \Vert ^2_{L^\infty }, \end{aligned}$$

which is precisely (3.16) after the usual change of variables. The proof is over. $\square$

## Appendix A: A General Result on Existence of Invariant Measures

The results in Sect. 3 on the existence of invariant measures can be stated in greater generality as follows. Let $(\mathcal {X}, d_s)$ be a metric space, and assume we are given another metric $d_w$ on $\mathcal {X}$ such that

1. Any $d_s$-bounded subset of $\mathcal {X}$ is $d_w$-precompact.
2. If $d_s(x_n,y_n)\rightarrow 0$ as $n\rightarrow \infty$ for some $x_n,y_n\in \mathcal {X}$, then $d_w(x_n,y_n)\rightarrow 0$ as $n\rightarrow \infty$.

We denote by $\mathcal {X}_w$ the space $\mathcal {X}$ endowed with the metric $d_w$. Given $x\in \mathcal {X}$, let $\{X_t^x\}_{t\ge 0}$ be a Markov process on $\mathcal {X}$ with $X_0^x=x$, and define the time-averaged measures

A.1 $$\begin{aligned} \mu ^x_T(B)=\frac{1}{T}\int _0^T{\mathbb {P}}(X_t^x\in B) \mathrm{d}t, \qquad T>0, \end{aligned}$$

where $B\in \mathcal {B}(\mathcal {X}_w)$, the Borel $\sigma$-algebra of $\mathcal {X}_w$. For $t\ge 0$, define the Markov semigroup

$$\begin{aligned} {\mathcal {P}}_t \phi (x)= {\mathbb {E}}\phi (X_t^x), \qquad \phi \in M_b(\mathcal {X}). \end{aligned}$$

We assume that the Markov semigroup is well defined on measurable bounded functions, namely the property that ${\mathcal {P}}_t : M_b(\mathcal {X}_w)\rightarrow M_b(\mathcal {X}_w)$ is well-defined.

### Definition A.1

Let $\phi \in M_b(\mathcal {X}_w)$. We say that $\phi :\mathcal {X}\rightarrow {\mathbb {R}}$ belongs to the class $\mathcal {G}$ if $\phi \in C(K)$ for any $d_s$- bounded set $K\subset \mathcal {X}$.

The following is a slight variant of the Krylov–Bogoliubov procedure to prove the existence of an invariant measure for ${\mathcal {P}}_t$.

### Theorem A.2

Assume that for some initial condition $x\in \mathcal {X}$ the following two properties hold.

- (Tightness) For every $\varepsilon \in (0,1]$, there exists a $d_s$-bounded and $d_w$-closed set $K_\varepsilon \subset \mathcal {X}$ such that the time-averaged measures $\{\mu ^x_T\}_{T>0}$ satisfy
  A.2 $$\begin{aligned} \inf _{T>0} \mu ^x_T(K_\varepsilon )\ge 1-\varepsilon . \end{aligned}$$
- (Almost Feller) For all $t>0$,
  A.3 $$\begin{aligned} {\mathcal {P}}_t C_b(\mathcal {X}_w)\subset \mathcal {G}. \end{aligned}$$

Then there exists an invariant measure $\mu$ for ${\mathcal {P}}_t$.

### Proof

From the tightness property (A.2) and the fact that $d_s$-bounded sets are $d_w$-precompact, we infer the existence of a sequence $T_j\rightarrow \infty$ and a measure $\mu \in {\mathfrak {P}}(\mathcal {X}_w)$ such that

A.4 $$\begin{aligned} \lim _{j\rightarrow \infty } \int _\mathcal {X}\phi \, \mathrm{d}\mu ^x_{T_j} = \int _\mathcal {X}\phi \, \mathrm{d}\mu , \qquad \forall \phi \in C_b(\mathcal {X}_w). \end{aligned}$$

Let $\varepsilon >0$, and let $K_\varepsilon$ be the corresponding sets as above. Now, since $K_\varepsilon$ is a $d_w$-closed set, the Portmanteau theorem and (A.2) imply

A.5 $$\begin{aligned} \mu (K_\varepsilon ) \ge \limsup _{T\rightarrow \infty }\mu _T(K_\varepsilon ) \ge 1-\varepsilon . \end{aligned}$$

We now claim that, in fact,

A.6 $$\begin{aligned} \lim _{j\rightarrow \infty } \int _\mathcal {X}\phi \, \mathrm{d}\mu ^x_{T_j} = \int _\mathcal {X}\phi \, \mathrm{d}\mu , \qquad \forall \phi \in \mathcal {G}. \end{aligned}$$

To prove the claim, let $\phi \in \mathcal {G}$ be arbitrarily fixed, and set

$$\begin{aligned} M_\phi :=\sup _{y\in \mathcal {X}} |\phi (y)|<\infty . \end{aligned}$$

It is important to notice here that $M_\phi$ does not depend on $\varepsilon$. We can then apply Tietze extension theorem (see e.g. [35]) and deduce the existence of a function ${\tilde{\phi }}_\varepsilon \in C_b(\mathcal {X}_w)$ such that

A.7 $$\begin{aligned} {\tilde{\phi }}_\varepsilon =\phi \quad \text {on } K_\varepsilon , \qquad \sup _{y\in \mathcal {X}}|{\tilde{\phi }}_\varepsilon (y)|\le M_\phi , \qquad \forall \varepsilon \in (0,1]. \end{aligned}$$

Now,

$$\begin{aligned}&\left| \int _{\mathcal {X}}\phi \, \mathrm{d}\mu ^x_{T_j}-\int _\mathcal {X}\phi \, \mathrm{d}\mu \right| \le \left| \int _{\mathcal {X}}(\phi -{\tilde{\phi }}_\varepsilon )\, \mathrm{d}\mu ^x_{T_j}\right| \\&\quad + \left| \int _{\mathcal {X}}{\tilde{\phi }}_\varepsilon \, \mathrm{d}\mu ^x_{T_j}-\int _\mathcal {X}{\tilde{\phi }}_\varepsilon \, \mathrm{d}\mu \right| + \left| \int _{\mathcal {X}}({\tilde{\phi }}_\varepsilon -\phi )\, \mathrm{d}\mu \right| . \end{aligned}$$

By (A.2) and the above (A.7), we have

$$\begin{aligned} \left| \int _{\mathcal {X}}(\phi -{\tilde{\phi }}_{\varepsilon })\, \mathrm{d}\mu ^x_{T_j}\right| =\left| \int _{\mathcal {X}\setminus K_\varepsilon }(\phi -{\tilde{\phi }}_{\varepsilon })\, \mathrm{d}\mu ^x_{T_j}\right| \le 2M_\phi \mu ^x_{T_j}(\mathcal {X}\setminus K_\varepsilon )< 2M_\phi \varepsilon . \end{aligned}$$

uniformly for $j\in {\mathbb {N}}$, and, similarly, in light of (A.5),

$$\begin{aligned} \left| \int _{\mathcal {X}}({\tilde{\phi }}_{\varepsilon }-\phi )\, \mathrm{d}\mu \right| <2M_\phi \varepsilon . \end{aligned}$$

Since ${\tilde{\phi }}_{\varepsilon }\in C_b(\mathcal {X}_{L^2})$, property (A.4) implies the existence of $j_\varepsilon \in {\mathbb {N}}$ such that

$$\begin{aligned} \left| \int _{\mathcal {X}}{\tilde{\phi }}_{\varepsilon }\, \mathrm{d}\mu ^x_{T_j}-\int _\mathcal {X}{\tilde{\phi }}_{\varepsilon }\, \mathrm{d}\mu \right| < 2M_\phi \varepsilon ,\qquad \forall j\ge j_\varepsilon . \end{aligned}$$

Hence, for every $\varepsilon >0$, there exists $j_\varepsilon \in {\mathbb {N}}$ such that

$$\begin{aligned} \left| \int _{\mathcal {X}}\phi \, \mathrm{d}\mu ^x_{T_j}-\int _\mathcal {X}\phi \, \mathrm{d}\mu \right| <6M_\phi \varepsilon , \qquad \forall j\ge j_\varepsilon , \end{aligned}$$

which proves (A.6). We are now left to prove that $\mu$ is invariant for ${\mathcal {P}}_t$. Let $\phi \in C_b(\mathcal {X}_w)$ be fixed. Thanks to assumption (A.3),

$$\begin{aligned} {\mathcal {P}}_t\phi \in \mathcal {G}, \qquad \forall t>0. \end{aligned}$$

In view of (A.6), we then have that

$$\begin{aligned} \lim _{j\rightarrow \infty } \int _\mathcal {X}{\mathcal {P}}_t\phi \, \mathrm{d}\mu ^x_{T_j} = \int _\mathcal {X}{\mathcal {P}}_t\phi \, \mathrm{d}\mu , \qquad \forall t\ge 0. \end{aligned}$$

Hence, by (A.1), we conclude that

$$\begin{aligned}&\int _\mathcal {X}{\mathcal {P}}_t\phi \, \mathrm{d}\mu \\&\quad =\lim _{j\rightarrow \infty } \int _\mathcal {X}{\mathcal {P}}_t\phi \, \mathrm{d}\mu ^x_{T_j} =\lim _{j\rightarrow \infty } \frac{1}{T_j}\int _0^{T_j} {\mathcal {P}}_{t+s} \phi (x)\mathrm{d}s =\lim _{j\rightarrow \infty } \frac{1}{T_j}\int _t^{T_j+t} {\mathcal {P}}_{s} \phi (x)\mathrm{d}s\\&\quad =\lim _{j\rightarrow \infty } \left( \frac{1}{T_j}\int _0^{T_j} {\mathcal {P}}_{s} \phi (x)\mathrm{d}s+ \frac{1}{T_j}\int _{T_j}^{T_j+t} {\mathcal {P}}_{s} \phi (x)\mathrm{d}s -\frac{1}{T_j}\int _0^{t} {\mathcal {P}}_{s} \phi (x)\mathrm{d}s\right) \\&\quad =\lim _{j\rightarrow \infty } \int _\mathcal {X}\phi \, \mathrm{d}\mu ^x_{T_j} = \int _\mathcal {X}\phi \, \mathrm{d}\mu , \end{aligned}$$

so that invariance follows from the arbitrariness of $\phi$. $\square$
